# Gold Nanoparticles Based Optical Biosensors for Cancer Biomarker Proteins: A Review of the Current Practices

**DOI:** 10.3389/fbioe.2022.877193

**Published:** 2022-04-26

**Authors:** Jinghua Tai, Shuang Fan, Siqi Ding, Lishen Ren

**Affiliations:** ^1^ Department of Gastroenterology, the Second Hospital of Jilin University, Changchun, China; ^2^ Department of Hematology and Oncology, The Second Hospital of Jilin University, Changchun, China

**Keywords:** gold nanoparticles, LSPR, SERS, optical biosensor, protein biomarker, plasmonic resonance

## Abstract

Cancer prognosis depends on the early detection of the disease. Gold nanoparticles (AuNPs) have attracted much importance in biomedical research due to their distinctive optical properties. The AuNPs are easy to fabricate, biocompatible, surface controlled, stable, and have surface plasmonic properties. The AuNPs based optical biosensors can intensely improve the sensitivity, specificity, resolution, penetration depth, contrast, and speed of these devices. The key optical features of the AuNPs based biosensors include localized surface plasmon resonance (LSPR), SERS, and luminescence. AuNPs based biomarkers have the potential to sense the protein biomarkers at a low detection level. In this review, the fabrication techniques of the AuNPs have been reviewed. The optical biosensors based on LSPR, SERS, and luminescence are also evaluated. The application of these biosensors for cancer protein detection is discussed. Distinct examples of cancer research that have a substantial impact on both scientific and clinical research are presented.

## 1 Introduction

The biological molecule that is present in blood or any body fluids or tissues and is an indicator of either normal or abnormal condition in the body is defined as a biomarker by the National Cancer Institute. Recently, biomarkers have achieved a lot of importance in the early diagnosis of cancer (Christofi et al., 2019). Cancer is a very serious disease that can lead to the death of a person if untreated (Yang et al., 2018). Survival can be made possible with the early diagnosis of the condition. It has been seen that in the case of early diagnosis of cancer, the therapy is given to have more effective and the recovery rate is much higher in such cases as compared to late diagnosed cases (Hawkes, 2019). The prognosis of cancer also depends upon the examination of the primary tumor and on the detection of disseminated cancer cells in case of a malignant tumor (Haber and Velculescu, 2014). Tissue biopsy is most commonly used for the clinical diagnosis of cancers, but it is very costly, time-consuming, invasive, unsuitable for repeated testing, and also unavailable in some cancers like lung cancer (Cui et al., 2018).

Liquid biopsy is a type of biopsy in which the analysis of biomarkers is done in the non-solid biological tissue such as blood. This method is quite effective and advanced and encircles a large number of diseases and cancer. Thus, this technique has many advantages over conventional ones (Alimirzaie et al., 2019). It is inexpensive, non-invasive, feasible for repeated testing, and available to a large population without any advanced requirements. It also allows disease monitoring, its stage, and real-time effectiveness of treatment as well (El Rassy et al., 2018; Rojalin et al., 2020). The circulating biomarkers like tumor cells, nucleic acids, vesicles, and proteins are all analytes that are analyzed in the liquid biopsy (Li et al., 2017; Lane et al., 2018; Chang et al., 2019).

In proteomics, some protein biomarkers have been investigated in blood for cancer diagnosis (Lewis et al., 2018). Elevated levels of proteins have been recorded in cancer patients which are co-related with the metastasis, prognosis, and treatment of the disease. Prostate-specific antigen (PSA) is one of the common protein biomarkers assessed in the clinical identification of prostate cancer (Tkac et al., 2019), Similarly, cancer antigen 125 (CA 125) level is checked for ovarian cancer (Romeo and Seropian, 2021), alpha-fetoprotein (AFP) level is checked for liver cancer (Zhang et al., 2018), CA19.9 level is checked for gastric/pancreatic cancer (Subki et al., 2021), carcinoembryonic antigen (CEA) level is checked for colorectal cancer (Zhao et al., 2018), and cancer antigen 15.3 (CA15.3)/CA27.29 level is checked for breast cancer (Li et al., 2018). Sometimes autoantibodies are also produced by the immune system in cancer patients. These autoantibodies are also used as biomarkers for some types of cancers as well. These autoantibodies most commonly include immunoglobulin G (IgG) and immunoglobulin E (IgE). IgG and IgE levels are usually high in cancerous patients as compared to normal persons (Zang et al., 2019). ELISA, western blotting, mass spectrometry, and radioimmunoassay (RIA) are some of the most often used techniques for protein biomarker analysis. These classical approaches, on the other contrary, are insensitive, time-consuming, or difficult to implement. Picomolar LOD values are typical among the commercially available immunoassays. However, the protein biomarker concentrations in the blood associated with various types of cancer show a range of 10^−16^–10^−12^ M in the blood. The low quantity of a given biomarker makes ELISA an ideal method for the detection of cancer proteins. There are a lot of cases where the biomarker is detected at an early stage of malignancy. But its application in hospital setting and as non-portable devices is not an ideal choice. As a result, it is imperative to create assays that are both sensitive and specific, as well as simple to use, for protein biomarker-based cancer diagnosis (Bakirhan et al., 2018).

In every aspect of daily science, nanotechnology has started new opportunities. Nanotechnology’s biological applications are progressing at a rapid pace to improve human health. The theragnostic platform for cancer detection is provided by nanocarriers (Ahmadian et al., 2019). Nanocarriers are one of a kind in terms of chemical solubilization, encapsulation, and disease marker detection capabilities. Among the numerous nanocarriers produced, gold nanoparticles (AuNPs) have garnered current scientific interest. Drug administration, tumor sensing, photothermal agents, and high-sensitivity biomarker testing are only a few of the uses of AuNPs (Medici et al., 2021). Other benefits of AuNPs are their simplicity of production, chemical stability, and biocompatibility.

This review focuses mainly on the surface plasmon properties of AuNPs for cancer treatment. The synthesis of AuNPs and important clinical biomarkers will be reviewed. The important physicochemical and optical properties of AuNPs and the techniques employed for cancer diagnosis will be discussed. The various strategies for detecting cancer protein biomarkers will be discussed in terms of their clinical application.

## 2 Synthesis of AuNPs

**FIGURE 1:**
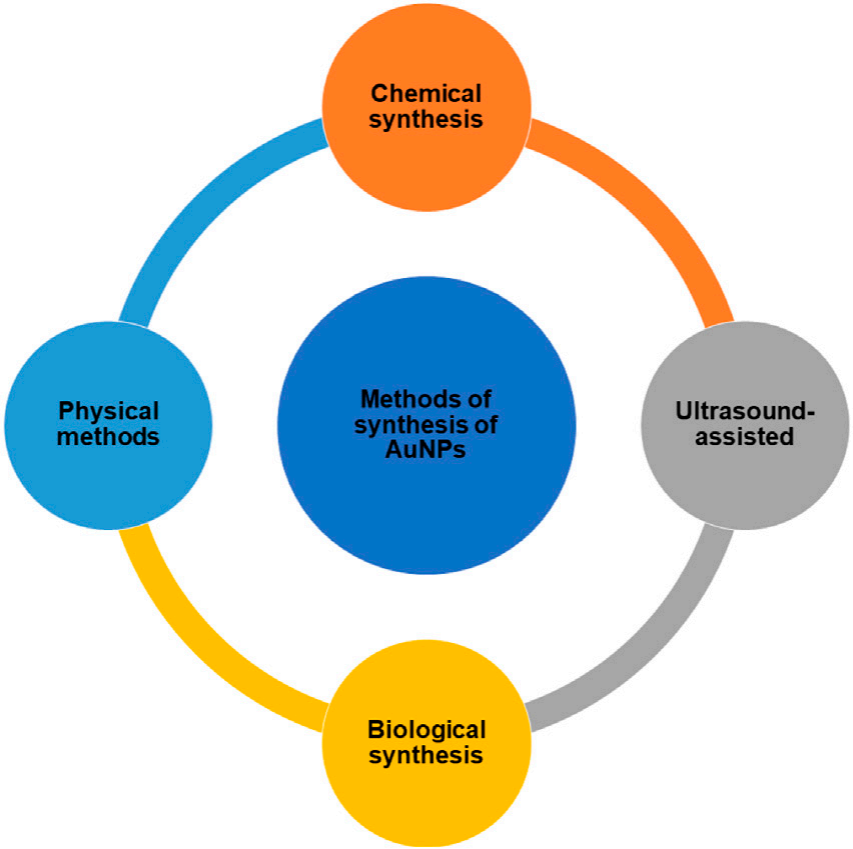
Methods for the synthesis of AuNPs.

AuNPs can be synthesized using “Top-Down” and “Bottom-Up” strategies, which are two distinct methods (Man et al., 2018; Wahab et al., 2018; Zhu et al., 2020). Starting with bulk material and breaking it down into nanoparticles using various ways is the top-down strategy to synthesize AuNP. The bottom-up strategy, on the other hand, begins with the atomic level and works its way up to the nanoparticle level. Top-down synthesis methods include laser ablation, ion sputtering, UV and IR irradiation, and aerosol technology, whereas bottom-up approaches include reducing Au^3+^ to Au^0^. The methods to synthesize the AuNPs can be classified as in Figure 1.

### 2.1 Chemical Synthesis of AuNPs

#### 2.1.1 Turkevich Method of Synthesis of AuNPs

**FIGURE 2:**
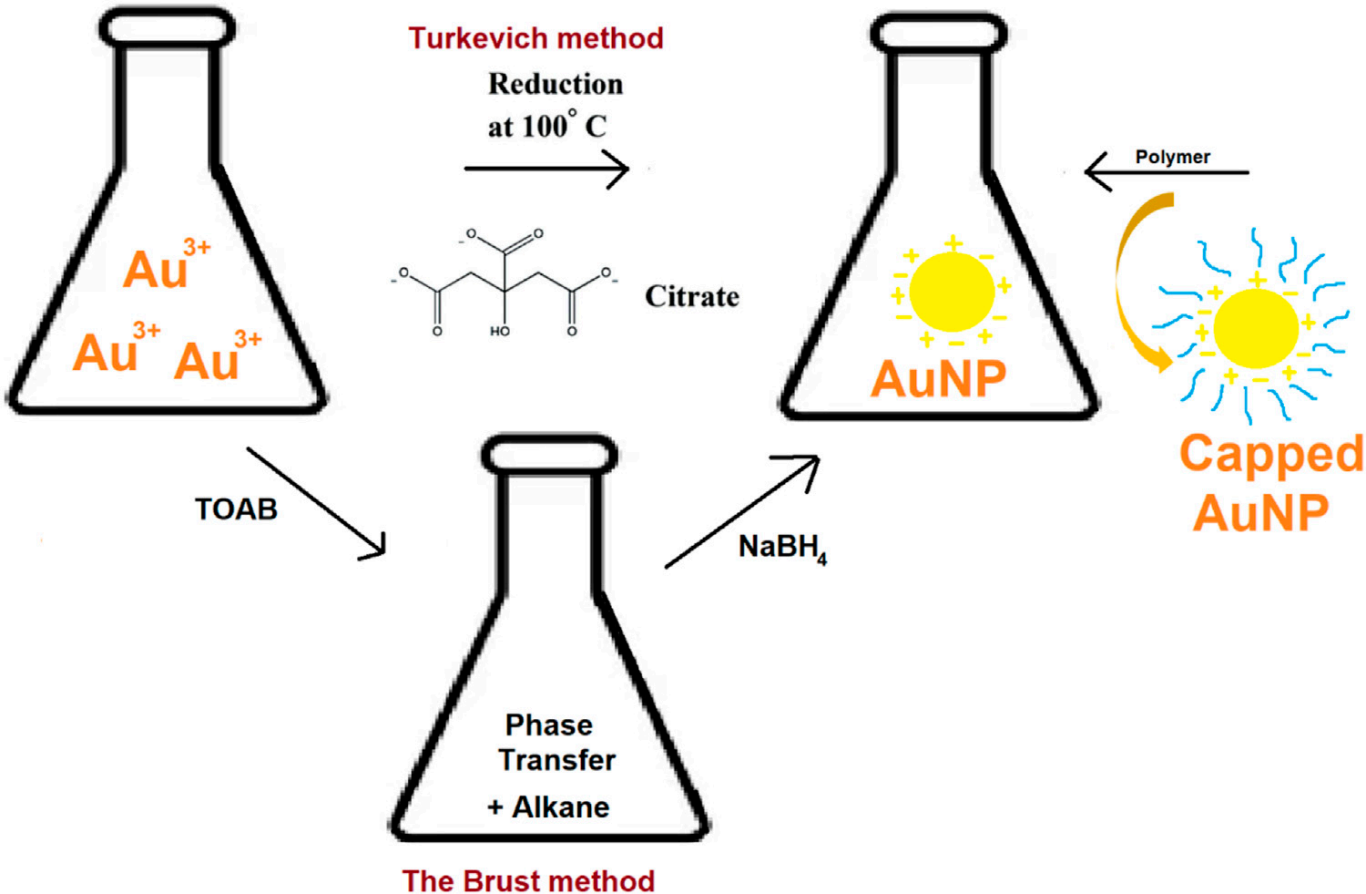
Turkevich method, Burst method and polymer capped synthesis of AuNPs.

Turkevich (1951) proposed the use of aqueous citrate solutions to reduce gold (III) compounds, such as HAuCl4, to produce AuNPs (Figure 2). The author’s experiments showed that the percentage of reducing to stabilizing agents has a significant impact on the size of the resulting nanoparticles (Polte et al., 2010). To produce AuNPs with many different shapes and sizes, borohydride was combined with chloroauric acid, which is a metallic salt of chloroauric acid. The varying amounts of the metal salt nanowires, nanorods, triangular and spherical type AuNPs were obtained (Grzelczak et al., 2008). It is also feasible to produce anisotropic metallic nanoparticles using an aqueous surfactant-based colloidal chemical approach in the presence of ascorbic acid and CTAB (Sau and Murphy, 2004). The AuNPs synthesis has been extensively done by using the classical Turkevich method. The synthesis of spherical particles between 10 and 30 nm has been carried out by this relatively reproducible and simple technique (Dobrowolska et al., 2015; Schulz et al., 2014).

#### 2.1.2 Polymer-Mediated Synthesis of AuNPs

In 1718, Helcher published a complete scientific article on polymer stabilized colloidal gold. According to numerous studies, metallic AuNPs and polymers have a considerable impact on particle size, stability, and distribution (Shenhar et al., 2005). Xinjiao et al., described a nanogold-based system successfully reduced to AuNPs by citrate and was encapsulated within the 154-block copolymer of polystyrene and poly (acrylic acid) (Figure 2). Such systems provide safe and effective diagnosis and targeted cancer treatment (Wang et al., 2008). Different polymers used in the synthesis of AuNPs include poly (ethylene glycol) (PEG) (Wang et al., 2020), Poly (N-vinylpyrrolidone) (PVP) (Fazleeva et al., 2021), chitosan (CS) (Hu et al., 2018), polystyrene (PS) (Gálvez-Vergara et al., 2020), poly (methyl methacrylate) (PMMA) (Wang and Seidel, 2021) and polyvinyl alcohol (PVA) (Amourizi et al., 2020).

#### 2.1.3 The Brust Method

Methods for synthesizing AuNPs employing organic solvents, have been documented since 1994. It produces AuNP within the size range of 1.5–5.2 nm. This method uses tetraoctylammonium bromide (TOAB) as a phase transfer (Figure 2) to transfer gold salt from aqueous solution to organic solvent (e.g., toluene) (Salabat and Mirhoseini, 2018). Sodium borohydride and an alkanethiol are then used to decrease the gold to a more reasonable size. The alkanethiol brings the stabilization of AuNPs. This process causes the color to change from orange to brown (Mahato et al., 2019), (Tianimoghadam and Salabat, 2018).

#### 2.1.4 Seed-Mediated Growth

**FIGURE 3:**
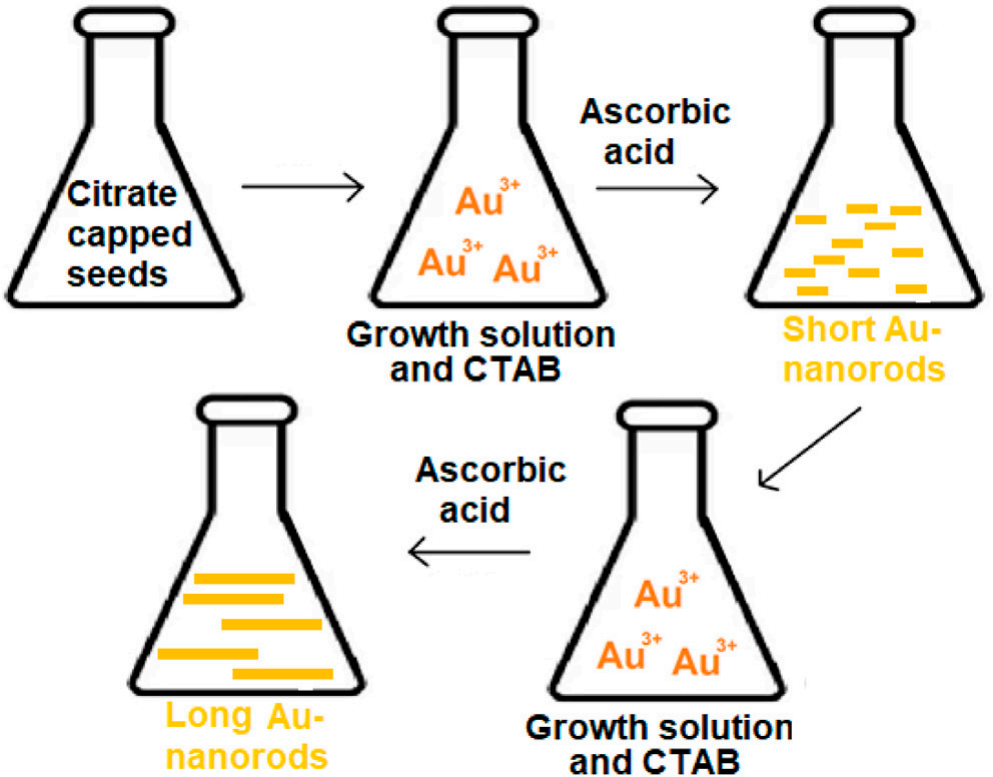
Schematic representation of seed mediated growth of AuNPs: long nanorods and short nanorods.

It is possible to formulate the AuNP in a variety of geometries and shapes such as rods and stars. Seed-mediated growth is the most frequent method for synthesizing rod-shaped AuNPs (Tianimoghadam and Salabat, 2018; Amini et al., 2018). This technique is based on the simple principle of generating seed particles from gold salts by lowering them to a lower concentration. Reducing agents like NaBH_4_ are used in this process. Then a metal salt and ascorbic acid is added to the seed particles to inhibit additional nucleation and speed up the synthesis of AuNPs of rod shape. The concentration of reducing agents and seeds affects the shape and geometry of gold nanoparticles (Cho et al., 2020), (Ramírez et al., 2020). A schematic representation of seed mediated growth of AuNPs is given in Figure 3.

### 2.2 Physical Methods for Synthesis of AuNPs

#### 2.2.1 UV-Induced Photochemical Synthesis of AuNPs

**FIGURE 4:**
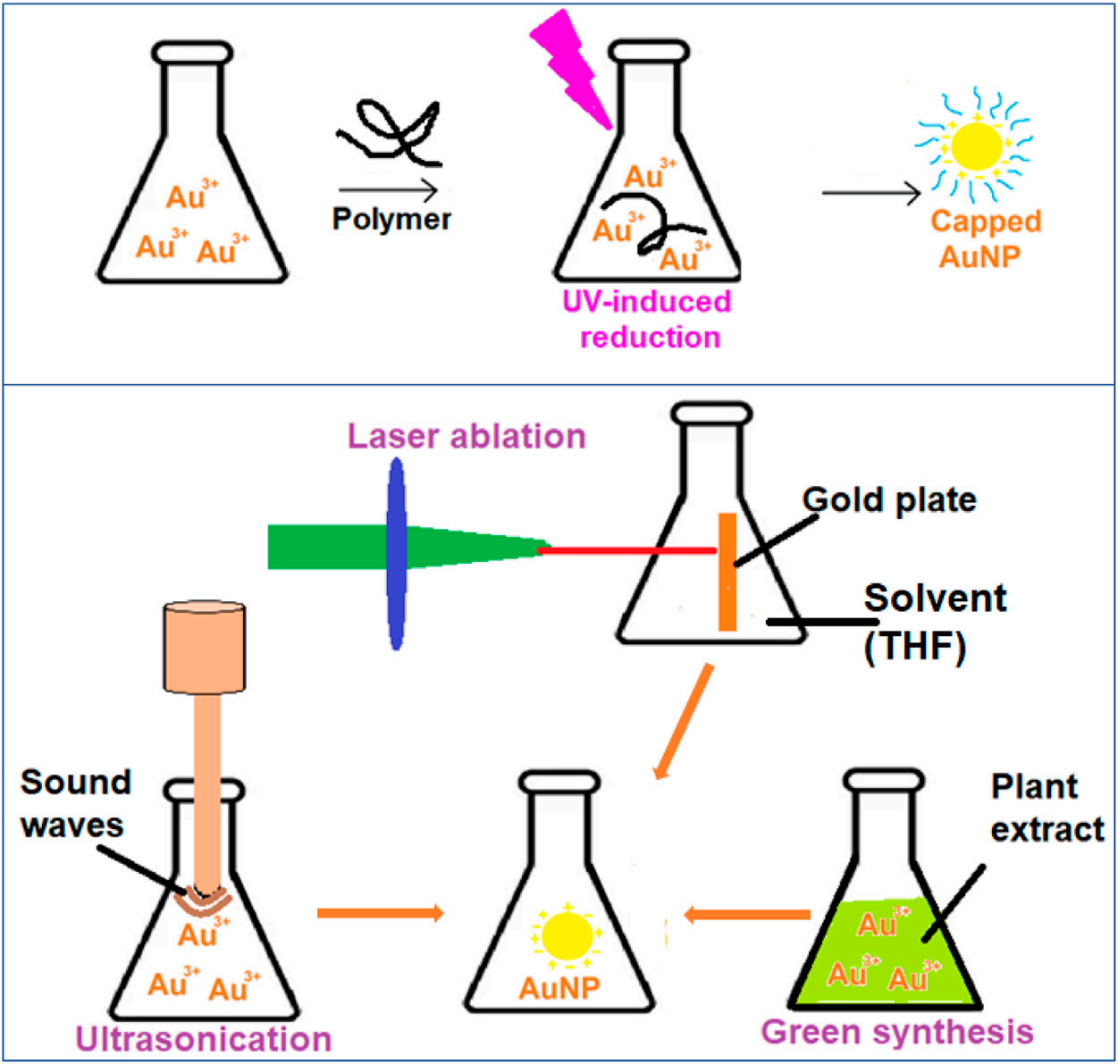
UV-induced, ultrasonication, laser ablation and plant extract based reduction schemes of AuNP synthesis.

Shengchun Yang et al. used photochemistry to successfully synthesize colloidal gold with a controlled size of nanoparticles (Zhou et al., 1999). AuNPs can be synthesized by employing the function of steric hindrance (Figure 4). The steric hindrance can be achieved by the capping effect of polymers, dendrimers, or surfactants as soft templates that prevent the formation of aggregates. The UV radiation source of various wavelengths, like Transilluminator 48 W light, and participation of surfactant/polymer reagents will have a specific impact on particle dimensions, which implies that raising the polymerization degree decreases particle size (Sau et al., 2001). In a study, Heparin functionalized colloids were generated using UV black-light lamp irradiation at approximately λ = 366 nm, and SERS (Surface Expanded Raman Spectroscopy) was carried out (del Pilar Rodríguez-Torres et al., 2014).

#### 2.2.2 Laser Ablation Synthesis of AuNPs

Recently, the reduction of gold (III) tetrachloroaurate metallic precursors by photo-induced effects of a 532 nm laser beam resulted in nanogold particles with diameters smaller than 5 nm. The gold is reduced to AuNPs in top-down strategy (Figure 4). Aqueous sodium dodecyl sulphate (SDS) solutions were used as a templating agent in this technique, and the scientists looked at the effects of SDS concentrations and laser influences on the produced AuNPs (Mafuné et al., 2001). In cancer biomarker applications, a two-step laser-assisted ablation technique was used to create surface enhanced Raman scattering (SERS) The laser-activated nano-rods include the bridging of connective tissue gaps (Matteini et al., 2010).

### 2.3 Ultrasound-Assisted Synthesis of AuNPs

When AuNPs are subjected to ultrasonication, the gold ions are reduced and form AuNPs (Figure 4). The characteristics of the particles can be adjusted. In one investigation, an ultrasound wave generator (200 kHz frequency, 20–200 W power output) was employed to maintain a consistent temperature for the ultrasonic-aided reduction of gold ions in a water bath while the presence of 2-propanol was also present. It was necessary to use a variety of stabilizers in the classic ultrasound-assisted synthesis process to assure repeatability and tunability. These included citrate, poly (N-vinyl-2-pyrrolidone), triphenylphosphine, disulfide, and several other dendrimers. Colloidal gold synthesis using an ultrasonic field is an intriguing and upfront method that may be applied in chemical and biological synthesis methods (Jiang et al., 2004)., (Okitsu et al., 2001)

### 2.4 Unconventional Green Biological Synthesis of AuNPs

Gold nanoparticles are typically prepared using chemical synthesis, which is a low-cost, high-volume process that yields reliable results (in terms of size and shape). However, the use of toxic solvents, contamination from precursor molecules, and deadly byproducts are some of the drawbacks to chemical synthesis (Shedbalkar et al., 2014). Modern nanotechnology research has turned its focus to biological synthesis to address these problems (Figure 4). Some of the biological sources are either found in nature or are revalued resources utilized during the creation of colloidal nanogold. These compounds include derivatives derived from plants, bacteria, fungi, algae, yeast, and viruses (Thakkar et al., 2010).

## 3 Characterization of AuNPs

### 3.1 LSPR, Size, Color and Electric Field Enhancement

**FIGURE 5:**
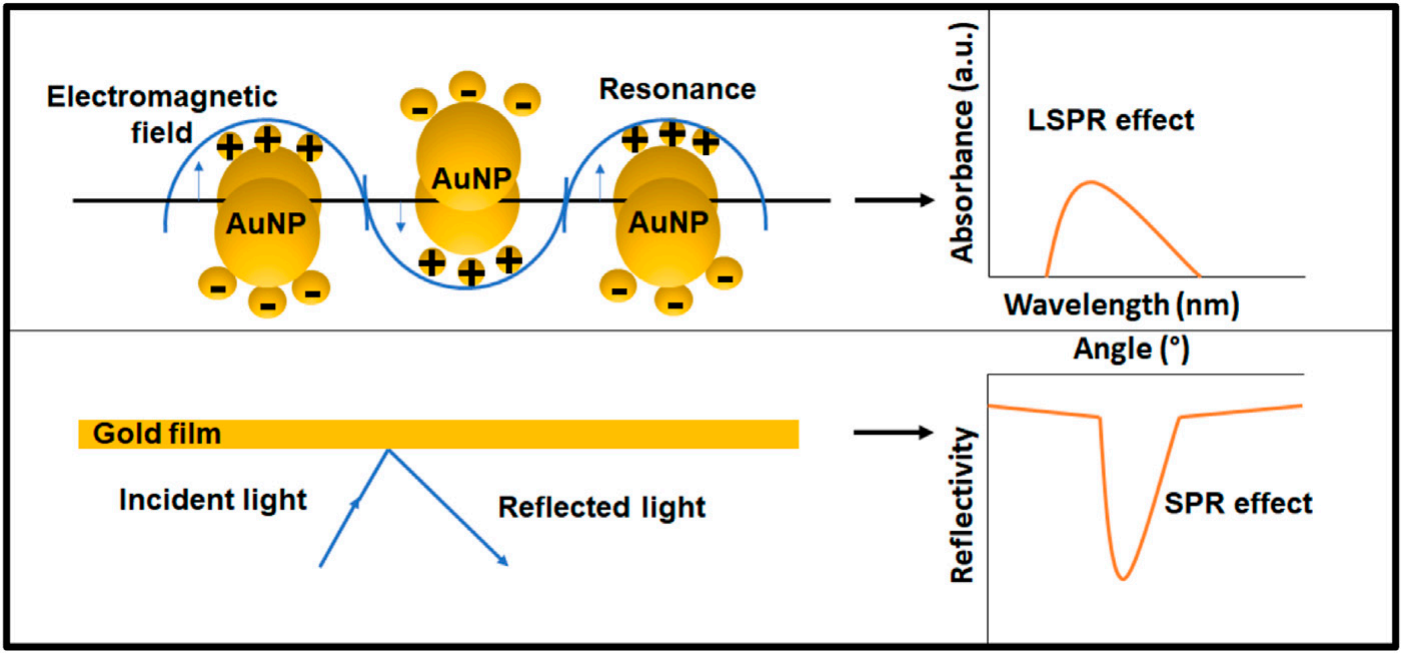
The principal of SPR and LSPR sensing techniques.

When the matter is exposed to light, numerous processes take place. Light may be absorbed. The scattering phenomenon occurs when light is dispersed. On the other hand, fluorescence occurs when light is re-emitted. Gold is a noble metal with many free electrons. When AuNPs are subjected to light, the electric field of the light causes the conduction-band electrons at the particle’s surface to collectively oscillate with respect to the ionic core of the nanoparticle (Huang et al., 2007). Concisely, the surface plasmon resonance is the coherent oscillation of metal-free electrons in resonance with the electromagnetic field (SPR). The SPR and LSPR are compared in Figure 5. The SPR equipment employed a monochromatic light source with a wide range of incident angles in the trials. As a result, the lowest point on the reflectivity vs. incident angle curve represents the plasmon excitation. It depicts that the coupling condition varies and the minimal peak shifts when the refractive index changes near the layer. For metal nanoparticles, the localized plasmons are excited by direct illumination, regardless of the illumination angle. The intensity of the transmitted light decreases because of the plasmon’s evanescent field surrounding the NP if the criterion on energy (the wavelength of the received light) is met. The abundance of readily polarized conduction electrons in AuNPs necessitates the use of nonlinear optical processes and electromagnetic fields as a preferred mode of interaction. LSPR has now been widely employed in AuNP based biosensors. Michael Faraday was the first to make the connection between AuNPs’ optical properties and their minuscule size at the Royal Institute in London. In his 1852 presentation entitled “Experimental Relations of Gold (and Other Metals) to Light,” the plasmonic properties were described. Traditional surface plasmon resonance (SPR) has been now replaced with localized surface plasmon resonance (LSPR). SPR makes use of gold film, while LSPR utilizes AuNPs. LSPR generates a prominent resonance absorbance peak in the visible region of light’s spectrum. AuNPs scatter light with an increase in intensity and at the same or a shifted frequency (elastic scattering-Resonance Rayleigh scattering) when light contacts their surface (inelastic scattering-surface enhanced Raman scattering). The location of this peak is highly dependent on the particle’s local refractive index. Instead of the angle seen in the traditional SPR method, the LSPR equipment can detect small variations in the wavelength of the absorbance location. LSPR offers numerous benefits, including the fact that it does not require a prism to combine the light, has a relatively short electromagnetic field decay duration, and is less susceptible to temperature drift. Sensor chips may be manufactured more cost-effectively (Focsan et al., 2017).

Gold nanoparticles absorb and scatter light with extraordinary efficiency. Their strong interaction with light occurs because the conduction electrons on the metal surface undergo a collective oscillation when they are excited by light at specific wavelengths (Cabuzu et al., 2015; Vines et al., 2019). In as study, the UV–visible spectra of the AuNPs samples were recorded for the wavelength range 200–800 nm at 2 min interval at 25°C on a Hewlett-Packard 8452A diode array spectrophotometer (Jiang et al., 2007). Gold nanoparticles represent specific wavelengths, emission frequencies, and emission wavelengths that are highly dependent on the size, shape, surface, and aggregation state of the nanoparticles (Kim and Lee, 2018). AuNPs with internal thiol groups on their metallic core were produced in a study. These demonstrated a UV-visible absorbance peak at 517 nm consistent with their surface plasmon resonance. These AuNP are not only soluble and stable in water, but also in different organic solvents such as dichloromethane or methanol (Ouellette et al., 2018). The characteristic optical properties and SPR of AuNP are summarized in Table 1.

**TABLE 1:** The characteristic optical properties of AuNPs.

Shape	Synthesis methodology	Size (nm)	Color	SPR (nm)	Ref
				(UV-visible λ_max_)	
Spherical	Laser ablation	17 and 32	-	536	Tommalieh et al. (2020)
Spherical	Green synthesis	between 20 and 50	Dark pink	537	Vijaya Kumar et al. (2019)
Semi-spherical	Turkevich method	10–13	Ruby red	529	Koushki et al. (2020)
Spherical	Polymer mediated	<50	Yellow to varying colors	520 to 540 of different systems	Jewrajka and Chatterjee, (2006)
Spherical	Green synthesis	5–20	Ruby red	520–550	Khatua et al. (2020)
Spherical	Green synthesis	25.5	Purple	530	Folorunso et al. (2019)
Nanospheres	Green synthesis	28.4	Red	532	Lee et al. (2019)
		190.7 (CS capped)		537 (CS capped)	
Nanostars		97.8	Bluish	800	
		123.9 (CS capped)		786 (CS capped)	
Nanorods	Growing nanorods from seeds	-	Pink	514 and 815	
				514 and 797 (CS capped)	
Nanorods	Growing nanorods from seeds	10	Yellowish	560	Qamar et al. (2022)

The changes in the pH have a drastic effect on the SPR of AuNPs especially in the case of functionalized nanoparticles. Researchers found that by varying the pH of AuNPs, the APTES assembly was controlled on the AuNP surface. This allowed for the reversible assembly of AuNPs. To monitor the optical behavior of the reversible assembly as a function of pH, a UV-vis spectrometer was utilized. In solution, the color changed from blue to bluish, followed by purple to wine red as pH increased from 9.8 to 12.0. The optical properties of AuNP assemblies showed an opposite phenomenon as pH decreased from 12.0 to 9.8 and the solution color changed from wine red, purple, and bluish purple tan (Liu et al., 2021). In metallic nanoparticles, LSPR causes resonances and enormous electric fields because of electric field resonance. Optical features of the structure have a greater effect on electric field enhancement than do material properties (Singh et al., 2013; Bensebaa, 2013). Yuan et al. prepared mercaptopyridine-functionalized AuNPs for fiber-optic surface plasmon resonance Hg^2+^ sensing. The basic principle was relied on the coordination between Hg^2+^ and nitrogen in the pyridine moiety. The electrical field intensity enhanced six times over the pure sensing gold film structure with the addition of AuNPs (Yuan et al., 2019).

### 3.2 X-Ray Crystallography (XRD)

Crystal structure and cell parameters are studied in relation to nanoparticle size and shape using XRD spectroscopy, a technique that can characterize nano powders of any size and shape (Vorontsov and Tsybulya, 2018). Van-Dat et al., Doan reported the biosynthesis of AuNPs Using *Litsea cubeba* Fruit Extract. XRD patterns were used to identify the crystalline structures of the biosynthesized AuNPs. There were distinct peaks in the XRD pattern of AuNPs at 2 theta angles of 38.12°, 44.27°, 64.42°, and 77.47°, which corresponded to the *hkl* planes of (111), (200), (220), and (311) respectively, for the face-centered cubic structure of Au (Doan et al., 2020). Bimetallic silver/gold nanoparticles were synthesized *via* starch-mediated synthesis. The Ag/AuNPs and AuNPs XRD spectrum indicated the bulk face-centered cubic (FCC) Ag and Au XRD patterns. In both bulk FCC Ag and Au, the diffraction peaks detected at 2 theta were 38.3, 44.5°, 64.8°, 77.8°, and 81.8° that corresponded to crystallographic planes (111), (200), (220), (311) and (222), respectively (Lomelí-Marroquín et al., 2019). Ehsan Koushki and co studied the effect of glucose and glucose oxidase on the UV–vis spectrum of AuNPs and reported the hkl planes of 111, 200, 220 and 311 in XRD spectrum (Koushki et al., 2020).

### 3.3 SERS and Electrical Field Enhancement

SERS is a surface-sensitive technique that works on amplified Raman scattering, when the molecules are adsorbed on rough metal surfaces or nanostructures (Bell et al., 2020). The electric field enhancement is the common mechanism involved in the SERS based metallic biosensors (Li et al., 2019). The surface plasmons are stimulated by the incident light. When the plasmon frequency, is in resonance with the radiation, the field enhancement is maximum (for spherical particles). The plasmon oscillations must be perpendicular to the surface for scattering to take place; if they are in plane with the surface, no scattering will take place (Palermo et al., 2021; Lu et al., 2020). The NP-derived nanostructured metal with SERS may deliver up to 3,000 times more enhancements in the electric field, which is equivalent to an increase in Raman scattering that is 14 orders of magnitude greater in magnitude. This improvement enables a single-molecule level of sensing. Jiajun Lu et al, investigated electric field-modulated SERS of metal-polymer hybrid. This technique improved the detection sensitivity (Lu et al., 2020). Zu-Yin Deng and co, synthesized gold and magnetic Fe_2_O_3_ nanoparticles that were sandwiched between layers of the biochip. Fe_2_O_3_ nanoparticles were used in the fabrication of single (Au/Fe_2_O_3_/Au) and multilayer (Au/Fe_2_O_3_/Au/Fe_2_O_3_/Au) chips to detect bovine serum albumin (BSA). The BSA antigen was detected with an SNR of 5.0 using a single-layer device. SERS was used to map the electromagnetic field enhancement. Using an external magnetic field, the single-layer chip containing Au nanoparticles was tested. In a field of 12.5 G, the strongest signal was detected. In a 62.5-G field, we found peaks caused by other carbon–hydrogen molecules (Deng et al., 2019). Due to its capacity to quench and multiplex fluorescence and its compatibility with short probe DNA sequences, SERS differs from conventional fluorescence-based techniques. Khalil et al., developed dual platform based on graphene oxide and AuNPs and a short DNA probe for SERS based DNA biosensing. The authors described a unique and PCR free SERS-based DNA detection technique for the identification of an endangered species, the Malayan box turtle (MBT), using dual platforms and short DNA probes (*Cuora amboinensis*). These two platforms were linked by covalently tying together graphene oxide-gold nanoparticles (GO-AuNPs) that were functionalized with capture probe 1 and AuNPs that were modified with capture probe 2 and a Raman dye (Cy3) *via* hybridization with the corresponding target sequences in order to detect them. Locally enhanced electromagnetic field “hot spots,” generated at junctions and interstitial fissures of nanostructures, enabled considerable amplification of SERS signals when the two platforms were coupled. As a result, the biosensors were able to reach a lower limit of detection (LOD) as low as 10 fM by employing two SERS active substrates and short-length probe DNA sequences. Even a single base mismatch in the target DNA could not fool the biosensor, which was also able to distinguish between six closely related non-target DNA sequences with high accuracy. That SERS biosensor was deemed fit to detect gene-specific biomarkers for numerous disorders, including cancer (Khalil et al., 2019). Two-dimensional (2D) gold nanoparticles can possess novel physical and chemical properties, which will greatly expand the utility of gold nanoparticles in a wide variety of applications ranging from catalysis to biomedicine. Muhammad alamri and co, developed plasmonic AuNPs on 2D MoS2/Graphene Vander-Waals heterostructures for high-sensitivity SERS. *In situ* electron-beam evaporation of Au at temperatures between 300° and 350°C in high vacuum resulted in the deposition of AuNPs on MoS2/graphene. As a SERS probe molecule, Rhodamine 6G (R6G) was shown to be five orders of magnitude more sensitive than AuNPs/graphene under the same excitation conditions, and this was done with a nonresonance 633 nm laser. Resonance 532 nm laser excitation resulted in a sensitivity increase from 5*10^−8^ M to 5*10^−10^ M. According to a density functional theory calculation, the observed increase in SERS sensitivity was attributed to the combination of the electromagnetic mechanism of plasmonic AuNPs with the chemical mechanism (Alamri et al., 2019).

## 4 Functionalization With Recognition Elements on AuNPs (Transducers)

The biosensors include both recognition elements and the transducer components, which work together to enable selective/specific binding with the target analytes to produce a signal. The AuNPs act as transducer and produce signal based on LSPR, SERS, chemiluminescence. Functionalization of NPs involves conjugation of molecules on the surface of the particles. The high surface to volume ratio allows efficient functionalization of particles to suit our needs (Thiruppathi et al., 2017). Table 2 includes some of the functional groups on the AuNPs. Farshchi and co., reported the polyethyleneimine-gold nanoparticles coupled to dye/metal ions electrochemical biosensor for the detection of three breast cancer biomarker proteins. Fe_3_O_4_ magnetic nanoparticles and cysteamine-functionalized AuNPs were used in the study to create a new electroconductive interface. In the electrochemical investigation of antibody-antigen binding, the constructed interface was used as a substrate for signal amplification. Anti-PSA antibody was bio-conjugated with Fe_3_O_4_ nanoparticles and drop-casted onto the surface of glassy carbon electrodes for this purpose (GCE). Secondary antibody (HRP-Ab2) encapsulated on AuNPs covered by cysteamine was fixed on the GCE modified electrode. Under ideal experimental circumstances, a detection limit of 0.001 µg.L^−1^ of PSA was achieved (Farshchi et al., 2020). In another study, Mahani et al., reported. The anti-PSA antibody was covalently coupled to the AuNPs. The antibody–PSA combination was also supported by MD simulations. Antigen binding caused the dielectric medium to change its refractive index, resulting in a shift in the LSPR peak of the probe. Accurate detection limits (0.2 ng ml^−1^) and calibration sensitivities of 43.75 nm/(ng mL^−1^)were achieved (Mahani et al., 2021).

**TABLE 2:** Functionalization of AuNPs.

Nano system	Functional group and ligand	Targeted application	Ref
AuNP	PEG Aminolated and Thiolated	Lateral Flow Detection of Bisphenol A	Lin et al. (2018)
Hollow Au Nanospheres and Au Nanorods	CLPFFD peptide- H_2_N-terminated thiol-PEG ligands	Inhibit Aβ-fibrillation	Ruff et al. (2018)
Methionine linked to AuNPs *via* dithiocarbamate	Amino acid (Methionine)	Tumor imagin	Gupta et al. (2021)
Composite phospholipid AuNPs	Folate	Tumor drug delivery and imaging	Sanzhakov et al. (2021)
pH responsive DNA-AuNPs	DNA/MUC1 aptamer	Anticancer	Chen et al. (2021)
AuNPs	Aromatic aminoacid	Lipid corona	Maity et al. (2021)

## 5 Clinical Protein Biomarkers for Early Cancer Detection

Detection and treatment of cancer are strongly dependent on early diagnosis. Tissue biopsy and traditional diagnostic methods such as ELISA have several drawbacks, including failure to identify at an early stage and the need for complicated procedures. Another method for diagnosing the existence of cancer and monitoring its progression is to screen for tumors using proteins contained in bodily fluids such as plasma, urine, and cells/tissues. In normal cells, these indicator proteins are produced at healthy levels; but, in a cancer environment, their levels rise by several orders of magnitude (Misek and Kim, 2011; Surinova et al., 2011; Nolen and Lokshin, 2012). ALK is a transmembrane tyrosine kinase receptor gene. During development, it is biologically expressed in the nervous system, but it vanishes after birth. Anaplastic large-cell lymphoma (ALCL) patients’ NPM1 fusion with ALK was first discovered in 1994. ALK has been linked to several malignancies, including non-small-cell lung cancer (NSCLC) (Du et al., 2018). The yolk sac and liver release the most alpha-fetoprotein (AFP), which is the most common blood protein in human embryos. In the mother’s blood, AFP levels peak between 28 and 32 weeks of pregnancy, decline rapidly after delivery and then spike again 8–12 months later. Adult AFP levels should not exceed 20 ng/ml. AFP is generated and secreted at a considerably higher rate in liver tumor cells (Shah et al., 2020). Among women, breast cancer is the most frequent invasive cancer and the second most common cause of cancer death after lung cancer. The clinical use of the cancer markers CA15-3, CA27-29, and Carcinoembryonic has been proven (EBSCOhost, 2021). Serum CEA, CA72-4, CA19-9, CA15-3, and CA12-5 are used to identify gastric cancer (Chen et al., 2017). KELIM, a reliable early indication of tumor chemosensitivity, was computed using CA-125 longitudinal kinetics over the first 100 treatment days in ovarian cancer (You et al., 2020). Similarly, a new ovarian cancer biomarker is transthyretin (TTR) and serum human epididymis protein 4 (HE4) (Zheng et al., 2018). In addition to its high prevalence and aggressiveness, bladder cancer is an extremely malignant tumor. Recurrence and death rates are very high for bladder malignancy. Early detection of bladder cancer and recurrences is essential to a long life expectancy. Several genetic variants are associated with a wide range of morphological indicators and clinical characteristics in bladder cancer. In the case of bladder cancer, fibrin/fibrinogen are important indicators. Thromboxin, IL-1, and IL-8 are upregulated in bladder cancer, posing a threat to the development of the tumor by interfering with blood vessel formation (Kwaan and Lindholm, 2019). CT screening for thyroid nodules can reveal medullary thyroid cancer (MTC) at an early stage by measuring both basal calcitonin (CT) and calcium (Ca)-stimulated CT levels (Niederle et al., 2020). Human IgG4-based Odronextamab is a first-in-class, hinge-stabilized, human IgG4-based bsAb (antibody). It has demonstrated good safety and tolerability, as well as early efficacy in patients with relapsed/refractory (R/R) B-cell non-Hodgkin lymphoma (B-NHL) (Bannerji et al., 2020). In men between the ages of 15 and 35, germ cell tumors (GCTs) are the most common type of cancer. Protein indicators such as Alpha-Fetoprotein (AFP) and free Beta HCG play an important role in pregnancy (Irfana Ishaq Sindhu et al., 2021).

## 6 AuNPs Based Optical Biosensing of Cancer Proteins

**FIGURE 6:**
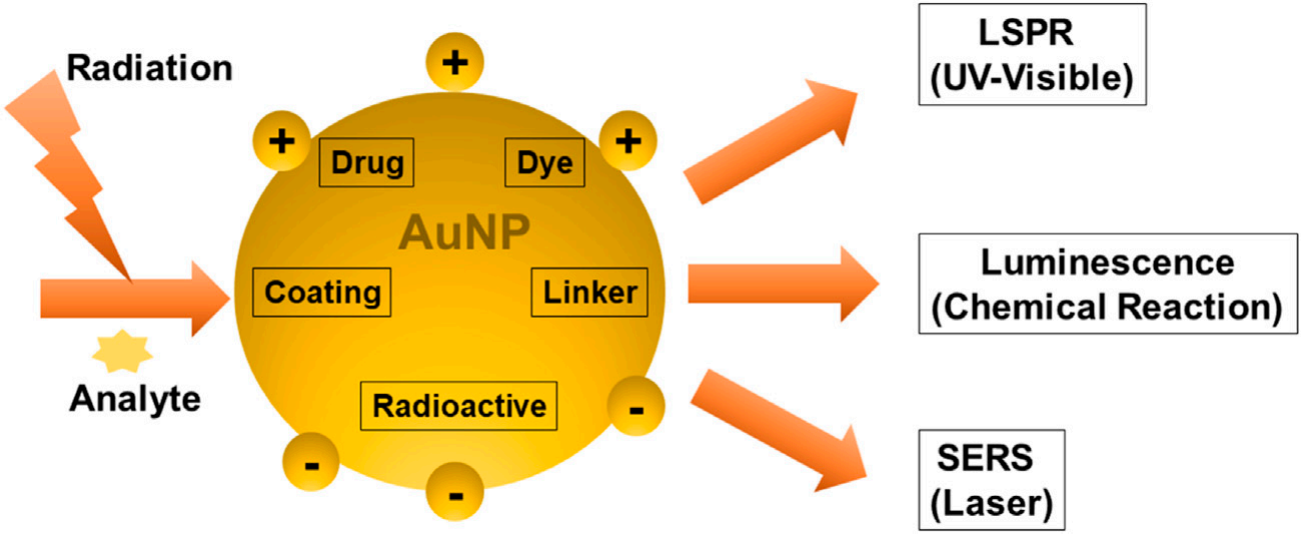
Techniques to analyze the Surface plasmon properties of multifunctional AuNPs.

The sensing mechanisms of optical biosensors that utilize AuNPs for cancer diagnosis, are based on LSPR, SERS, and chemiluminescence. LSPR is an optical phenomenon that occurs when light photons interact with AuNP’s conduction band. It results in the collective oscillation of valence electrons and absorption in the ultraviolet-visible (UV-Vis) band. When a laser is used as the light source, the technique used is Raman spectroscopy. The SERS technique works by amplifying the Raman response of an analyte when it interacts with the surface plasmon of metals such as AuNPs. Luminescence is a broad term that refers to the emission of light that is not caused by a high temperature. Chemiluminescence is a light-emitting technology that is based on an exciting intermediate in a chemical reaction. When this intermediate reaches the ground state, it emits light. In contrast to fluorescence, electrons in chemiluminescent materials are activated *via* a chemical reaction rather than photon absorption. Surface plasmon resonance gives AuNPs adjustable optical characteristics (Figure 6).

### 6.1 Optical Biosensing Based on SPR/LSPR-Enhanced Absorption

**FIGURE 7:**
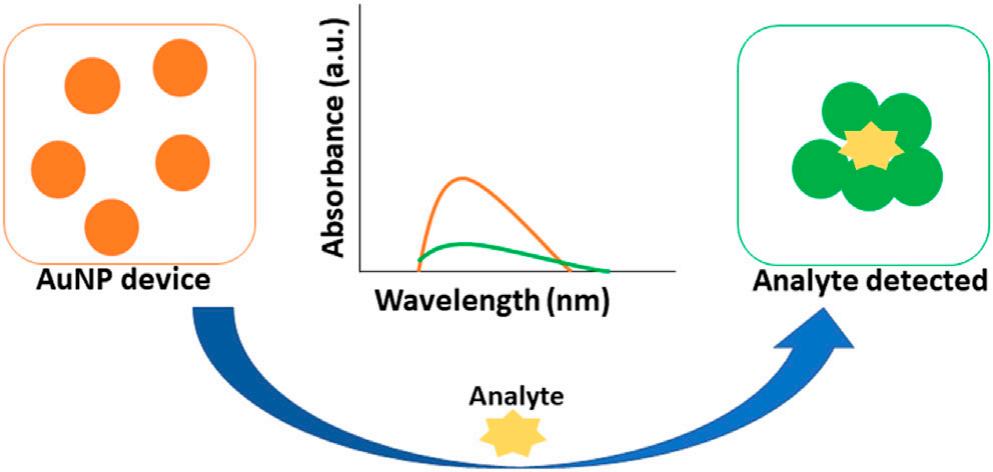
AuNP based colorimetric LSPR.

Colorimetric biosensing and Refractive index-based biosensing are the sensing mechanisms involved in LSPR devices. The small changes in refractive index are measured and the color changes are observed. The colorimetric analysis is a method that compares the color variations of a solution to determine the concentration of the analyte. There is no need for a fluorescent label in the case of a refractive index. For cancer protein biomarkers, AuNPs revealed a single LSPR shift as a signal reading. AuNPs are utilized to diagnose bladder cancer, according to Jazayeri and others. The antibody was detected using anti-survivin-coated AuNPs, and the findings were compared to ELISA. The AuNPs coupled with an anti-survivin antibody can detect bladder cancer in its early stages (Jazayeri et al., 2020). The citric acid reduction technique was used by Mahani et al. to make simple and cost-effective AuNP. These nanoparticles were able to identify prostate cancer in its early stages. The ultra-sensitive label-free nano-biosensor was created to measure PSA in serum. It could detect PSA at low concentrations of 0.2 ng ml^−1^. Based on the hydrogen bonding between PSA and antibody, the LSPR method was utilized (Mahani et al., 2021). Retout et al. described the preparation of peptide-functionalized AuNP using a colorimetric LSPR-based fast and selective protein detection technique. At concentrations as low as 20 nM, the system proved effective, with signals appearing in less than 5 min (Retout et al., 2016). Kim and his colleagues created the refractive index sensor utilizing AuNP-based optical fibers. AuNPs are a kind of nanoparticle that attaches to the optic fiber and improves its stability. Thyroglobulin is an immunoglobulin that has been identified as a thyroid cancer biomarker with a LOD of 0.19 pg/ml. The data were examined to see if the biomarker might be detected in cancer patients’ sera (Kim et al., 2019). Figure 7 depicts the visual color changes in AuNP based LSPR for the detection of small biological molecules and cells.

### 6.2 Optical Biosensing Based on SERS

**FIGURE 8:**
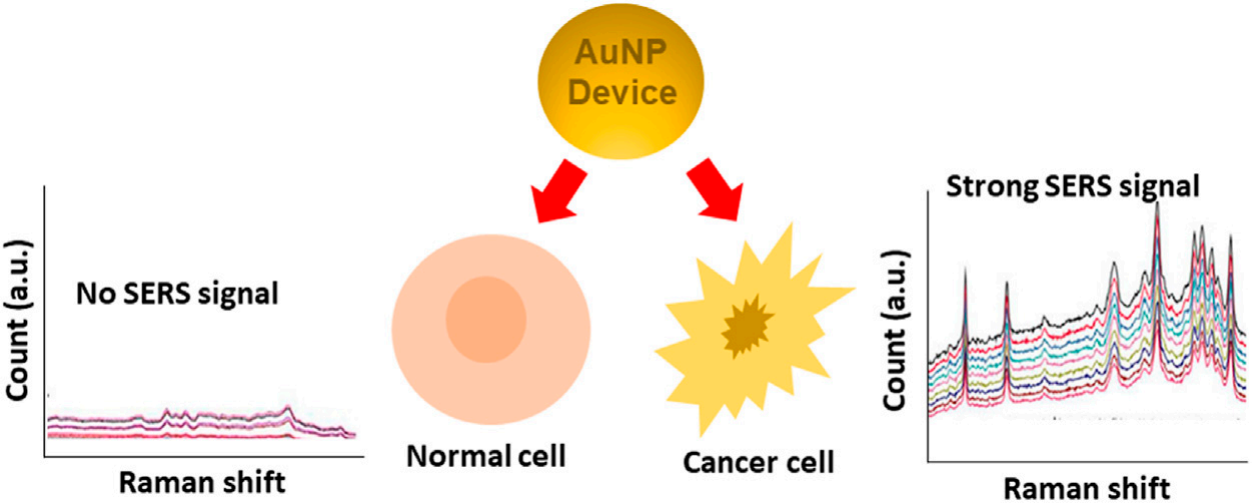
AuNP based SERS for metastatic cancers diagnosis.

AuNPs can be used as protein biosensors employing surface-enhanced Raman spectroscopy (SERS), also known as surface-enhanced Raman scattering. As a surface-sensitive method, it has the potential to identify single molecules (Blanco-Formoso and Alvarez-Puebla, 2020). It utilizes molecules adsorbed on rough metal surfaces or nanostructures like plasmonic AuNPs. A molecule’s vibrational spectrum can be boosted by several orders of magnitude with this method (Moskovits, 2005). Direct and indirect methods of SERS sensing are the two main sensing mechanisms involved in these devices. In direct sensing, an analyte molecule is directly attached to a plasmonic substrate, and the vibrational modes of the analyte are reflected in the wavelengths of the resulting spectral characteristics. Rather than the analyte itself, the SERS signal is obtained using a reporter molecule (usually a dye or another significantly Raman scattering molecule. Due to the non-linearity of SERS enhancement at high analyte concentrations, the adsorption of molecules onto nanoparticle surfaces is not always uniform, and inefficient “hot spot” formation due to large particle spacing results in decreased enhancement. So, it can be difficult to correlate spectral intensity to concentration when direct sensing is used. Indirect sensing approaches can circumvent this drawback by selectively capturing analyte molecules and bringing them close to the augmenting surface using capture ligands, molecular recognition agents, or antibodies. However, because indirect sensing does not directly probe the molecule of interest, we are unable to learn about the molecule’s vibrational modes (Moore et al., 2018). SERS and hollow-core photonic crystal fiber (HCPCF) were employed by Dinish and others to detect protein biomarkers with exceptional sensitivity. For the nanoprobe, powerful Raman active molecules were attached to the surface of AuNPs, which can be used in conjunction with recognition element species to identify specific molecules. The biosensor was put to the test against MCF, a breast cancer cell line, and compared to an ELISA. In a sample volume of 10 nL, the nano-system was able to identify 100 pg of proteins (Dinish et al., 2012). Similarly, to explore the link between malignant exosomes and protein markers for cancer diagnosis, shin et al., used SERS principal component analysis (PCA). Cells from the PC9 and H1299 strains were used to investigate the effect. For this study, the researchers looked at the Raman peaks of four different exosomal protein identifiers: CD9, CD81, EpCAM, and EGFR (Shin et al., 2018). Biomarkers for lung and breast cancer treatment included elevated levels of IL-6 in the serum of patients. Inflammatory and cancer-promoting cytokine interleukin-6 (IL-6) was recently discovered by Muhammad et al., in a label-free, delicate, and selective biosensor. The IL-6 recognition sequence was incorporated into an aptamer on the SERS substrate, which was functionalized with an output signal reporter and an Au NPs array. When measuring IL-6 levels, SERS was utilized instead of ELISA. The 10^−12^–10^−7^ M range of IL-6 was successfully quantified. A biomedical application for the SERS-based aptamer biosensor has been demonstrated (Muhammad et al., 2021). Figure 8 presents SERS based probe device of AuNPs for cancer detection. The SERS based system has the potential to detect the malignancy signals from various cancer cell lines.

### 6.3 Optical Biosensing Based on AuNP-Induced Luminescence

**FIGURE 9:**
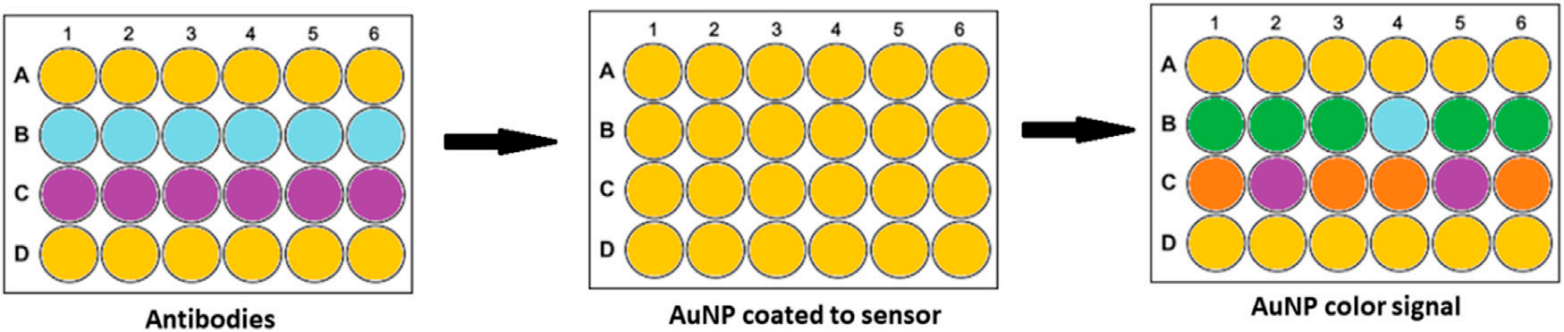
Chemiluminescence in AuNP immunoassay for cancer biomarker detection.

The Luminescence-based optical biosensors use either fluorescence or chemiluminescence for detection. It offers a wide range of advantages i.e., increased selectivity and flexibility. The colorimetric type fluorescence-based biosensors give visual results by using AuNP fluorescence or fluorescence resonance energy transfer (FRET) (Elahi et al., 2019), (Saeed et al., 2017). Zhang and others created ultrasensitive electrogenerated chemiluminescence (ECL) biosensor for exosomes and their surface proteins by forming AuNPs adorned Ti3C2 MXenes hybrids with aptamer modification *in situ* (AuNPs-MXenes-Apt). Exosomes were efficiently collected using this method using an exosome-recognized CD63 aptamer modified electrical interface. A highly sensitive ECL biosensor for the detection of exosomes contributed to the synergistic effects of increased surface area, exceptional conductivity, and catalytic activity of AuNPs-MXenes-Apt. The detection limit for exosomes obtained from HeLa cell lines is 30 particles µL -1 which was 1,000 times lower than the conventional ELISA method. The ECL sensing platform has high selectivity towards exosomes and surface proteins obtained from different kinds of tumors (OVCAR, HeLa, and HepG2 cells). It also facilitated the sensitive and precise detection of exosomes from human serum which depicts that the ECL biosensor is a viable, sensitive, and reliable tool for the detection of exosomes in clinical diagnostics (Zhang H. et al., 2020). For the detection of α-fetoprotein (AFP), a proximity hybridization-regulated electrogenerated chemiluminescence bioassay was developed *via* gold nanoparticles (AuNP) sensitization and target-induced quenching mechanism (Gao et al., 2017). The PLA-ECL bioassay was revealed to be more rapid, efficient selectivity, easy operation, precise accuracy, and wide detachable range, which are favorable for the detection of AFP in serum samples. The detachable limit for AFP was found to be 0.04 ng/ml. The bioassay can detect 300 a.m. (∼9,000 copies) of prostate-specific antigen in buffer and 3 fM in 10% serum. Moreover, three protein cancer markers were detected at low pM concentration of buffer and 10% serum. Figure 9 demonstrates the application of AuNPs based on the principle of chemiluminescence.

## 7 Clinical Applications of Cancer Proteins Based AuNP Biomarkers

**FIGURE 10:**
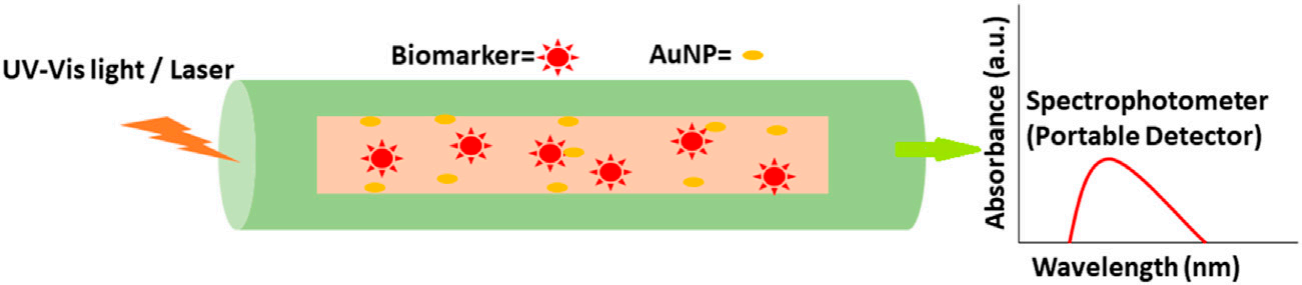
AuNP fabricated portable optic fiber biosensor in cancer biomarker detection.

AuNPs can improve conventional tests by improving sensor-analyte interactions. For cancer proteins, the detection limit (LOD) was increased by using AuNPs on ELISA plates (Ambrosi et al., 2010). UV-Vis spectroscopy may be used to determine the amounts of Au NPs in the presence and absence of proteins (Kuntamung et al., 2021). AuNPs-based bio-barcode assays were found to be even more sensitive than ELISA-based procedures without enzymatic amplification. Using immunomagnetic beads, magnetic separation can be utilized to isolate proteins from aqueous solutions. Immunomagnetic beads and AuNP conjugates are placed between the proteins. After the magnetic separation, the barcode oligonucleotides are rehybridized and then detected with an Au-NP catalyzed silver enhancement chip-based scano-metric assay. PSA may be detected in PBS at attomolar (10^−18^ M) concentrations using this method (Nam et al., 2003). Optic microfiber-based biosensors are likewise getting better and better (Figure 10). A microfiber optic biosensor was developed for detecting evanescent wave absorption as a cancer diagnostic. The biosensor’s microfiber surface was coated with an anti-AFP capture antibody. Secondary antibodies containing AuNPs were used as signal amplifiers. This drop-in light output can be attributed to the extraordinary absorption capacity that AuNPs have. It was only when AuNPs neared the fiber surface and preferentially absorbed evanescent waves that the biomarker was identified. AuNPs in optical sensors considerably increased sensitivity by signal amplification of refractive index changes/EW absorption phenomenon changes, rapid detection time, high resolution, and real-time label-free sensing. Although a greater theoretical understanding of the plasmonic shaping in AuNPs, innovative gold nanostructures and nanohybrid structures, dielectric media RI and nanostructured material production are needed for future ultrasensitive plasmonic nano-sensors for point-of-care (POC) applications (Li et al., 2014).

SERS enhancement on the high curvature tips and edges of anisotropic Au NPs, such as NSTs, has recently been employed to increase detection sensitivity. SERS Au NSTs (malachite green isothiocyanate as the Raman reporter) and an Au triangular nanoarray were utilized to develop a novel test with great sensitivity. By trapping sandwich nanoparticles over the gold triangular nanoarray and increasing the electromagnetic field intensity and 3D space, protein biomarkers produce a limited 3D plasmonic environment. Raman reporter molecules are exposed to multiple “hot spots” in the SERS immunosensor, which dramatically amplifies the Raman signal. The linear range of this SERS immunosensor for human immunoglobulin G protein in the buffer solution is 0.1 pg/ml to 10 ng/ml, and the detection limit is low (7 fg/ml) (Li et al., 2013). Table 3 lists some of the AuNPs-based optical biosensors that have been developed as cancer protein biomarkers.

**TABLE 3:** AuNPs-based optical biosensors as cancer protein biomarkers.

Protein marker	Cancer type	Optical techniques	Sensitivity lower limit/Linear range	References
CEA	Liver, ovarian, testicular	SERS	0.0001–100.0 ng ml^−1^	Medetalibeyoglu et al. (2020)
HER2	Breast, gastric, esophageal	Chemiluminescence	-	Zhang P. et al. (2020)
SCC	Esophageal, lung, ovarian	FDG-PET	-	Peng et al. (2020)
PSA	Prostate cancer	LSPR	100 fg/ml	Sanders et al. (2014)
PSA	Prostate cancer	SERS	1 pg/ml	Grubisha et al. (2003)
PSA	Prostate cancer	LSPR- Quenchers/enhancers	0.032 pg/ml	Liu et al. (2013)
AFP	Liver, ovarian, testicular	LSPR	93.11 fg/ml	Kim et al. (2021)
CA19-9	Bile duct, gastrointestinal	Luminescent	0.007 U ml^−1^	Alarfaj et al. (2018)
CA72-4	Bile duct, gastrointestinal	Chemiluminescence	-	Hu et al. (2019)
Osteocalcin	Bone	Luminescent	-	Matsuyama et al. (2018)
CYFRA 21–1	Lungs	Luminescent	0.08–500 ng/ml	He et al. (2013)
TPA	Lungs	SERS	-	Hong and Li, (2013)
5-HIAA	Carcinoid	SERS	1.2 ng/ml/12 min	Zhang Y. et al. (2020)
NMP 22	Bladder	Luminescent	0.05 pg ml^−1^	Othman et al. (2020)
PSA	Prostate	SERS	0.1 μg.L^−1^	Ouhibi et al. (2021)
S100	Melanoma	SERS	0.0001 ng/ml	Song et al. (2022)
Thyroglobulin	Thyroid	LSPR	-	Kim et al. (2021)
CA27.29	Breast	SERS	CA15-3 (0.99 U ml^−1^), CA27-29 (0.13 U ml^−1^) and CEA (0.05 ng ml^−1^)	Li et al. (2015)
CA15-3	Breast	Luminescent	39.2 and 89 μu.ml^−1^ for CA15/and CA72-4/respectively	Asbaghian-Namin et al. (2021)
Ferritin	Liver	SERS	0.41 pg/ml	Ma et al. (2020)
HE4	Ovarian	SERS	10^−17^ M	Eom et al. (2021)
CA125	Ovarian	SERS	-	TunÇ and Susapto, (2019)
Apolipoprotein A1	Ovarian	Luminescent	-	García et al. (2021)
BRAF V600E	Colon	SERS	1–5,000 fmol	Yu et al. (2020)

## 8 Conclusion and Perspectives

The discovery of peripheral biomarkers that are linked to cancer treatment grows as the disease progresses. Tumor cells, vesicles, nucleic acids, and proteins are all examples of this. The detection and prognosis of cancer patients can be improved by using biomarkers that circulate in the bloodstream. As a result, better detection methods are critical. Flow cytometry, ELISA, and DNA and protein arrays, among other standard analytical methods, have played a critical role in this field’s advancement. Because of a lack of sensitivity, they are ineffective for cancer screening and diagnosis. Biomarker classification has been made easier thanks to the wide variety of nanotechnology-based approaches that have been developed. It has been possible to develop several other tests using AuNPs, including those that use LSPR and colorimetry in addition to SERS and fluorescence. There are numerous uses for each of them, depending on the goal and the technique of reading the signal. AuNP-based optical sensing systems can be used in mobile, wearable, and even implantable devices. For long-term sustainability, AuNPs must be established on a broad scale and in a variety of environments. Reduced analysis time and prevention of non-specific adsorption of biomolecules onto AuNPs are two further ways to improve the analytical performance of biosensors. To better understand the biodistribution and clearance of AuNPs used in biomedical imaging, more research is needed. Additionally, new targeting agents and targeting strategies are needed to improve molecular imaging’s specificity. Investigating the creation of AuNP composites or hybrids with other nanoparticles is vital to discovering the potential for new research and application prospects. Multifunctional AuNPs and nanohybrids based on AuNPs are likely to be increasingly popular in the near future in relation to multi-analyte imaging.

## References

[B1] Ahmadian E. Dizaj S. M. Sharifi S. Shahi S. Khalilov R. Eftekhari A. (2019). The Potential of Nanomaterials in Theranostics of Oral Squamous Cell Carcinoma: Recent Progress. Trac Trends Anal. Chem. 116, 167–176. 10.1016/j.trac.2019.05.009

[B2] Alamri M. Sakidja R. Goul R. Ghopry S. Wu J. Z. (2019). Plasmonic Au Nanoparticles on 2D MoS2/Graphene van der Waals Heterostructures for High-Sensitivity Surface-Enhanced Raman Spectroscopy. ACS Appl. Nano Mater. 2 (3), 1412–1420. 10.1021/acsanm.8b02308

[B3] Alarfaj N. A. El-Tohamy M. F. Oraby H. F. (2018). CA 19-9 Pancreatic Tumor Marker Fluorescence Immunosensing Detection via Immobilized Carbon Quantum Dots Conjugated Gold Nanocomposite. Int. J. Mol. Sci. 19 (4). 10.3390/ijms19041162 PMC5979385 29641488

[B4] Alimirzaie S. Bagherzadeh M. Akbari M. R. (2019). Liquid Biopsy in Breast Cancer: A Comprehensive Review. Clin. Genet. 95 (6), 643–660. 10.1111/cge.13514 30671931

[B5] Ambrosi A. Airò F. Merkoçi A. (2010). Enhanced Gold Nanoparticle Based ELISA for a Breast Cancer Biomarker. Anal. Chem. 82 (3), 1151–1156. 10.1021/ac902492c 20043655

[B6] Amini A. Kamali M. Amini B. Najafi A. (2018). Enhanced Antibacterial Activity of Imipenem Immobilized on Surface of Spherical and Rod Gold Nanoparticles. J. Phys. D: Appl. Phys. 52, 065401. 10.1088/1361-6463/aaef4d

[B7] Amourizi F. Dashtian K. Ghaedi M. (2020). Polyvinylalcohol-citrate-stabilized Gold Nanoparticles Supported congo Red Indicator as an Optical Sensor for Selective Colorimetric Determination of Cr(III) Ion. Polyhedron 176, 114278. 10.1016/j.poly.2019.114278

[B8] Asbaghian-Namin H. Karami P. Naghshara H. Gholamin D. Johari-Ahar M. (2021). Electrochemiluminescent Immunoassay for the Determination of CA15-3 and CA72-4 Using Graphene Oxide Nanocomposite Modified with CdSe Quantum Dots and Ru(bpy)3 Complex. Microchim. Acta 188 (7), 1–13. 10.1007/s00604-021-04890-2 34184115

[B9] Bakirhan N. K. Ozcelikay G. Ozkan S. A. (2018). Recent Progress on the Sensitive Detection of Cardiovascular Disease Markers by Electrochemical-Based Biosensors. J. Pharm. Biomed. Anal. 159, 406–424. 10.1016/j.jpba.2018.07.021 30036704

[B10] Bannerji R. Allan J. N. Arnason J. E. Brown J. R. Advani R. Ansell S. M. (2020). Odronextamab (REGN1979), a Human CD20 X CD3 Bispecific Antibody, Induces Durable, Complete Responses in Patients with Highly Refractory B-Cell Non-hodgkin Lymphoma, Including Patients Refractory to CAR T Therapy. Blood 136 (Suppl. 1), 42–43. 10.1182/blood-2020-136659

[B11] Bell S. E. J. Charron G. Cortés E. Kneipp J. Chapelle M. L. Langer J. (2020). Towards Reliable and Quantitative Surface‐Enhanced Raman Scattering (SERS): From Key Parameters to Good Analytical Practice. Angew. Chem. Int. Ed. 59 (14), 5454–5462. 10.1002/anie.201908154 PMC7154527 31588641

[B12] Bensebaa F. (2013). Optoelectronics. Interf. Sci. Technol. 19, 429–479. 10.1016/b978-0-12-369550-5.00007-0

[B13] Blanco-Formoso M. Alvarez-Puebla R. A. (2020). Cancer Diagnosis through SERS and Other Related Techniques. Int. J. Mol. Sci. 21 (6), 2253. 10.3390/ijms21062253 PMC7139671 32214017

[B14] Cabuzu D. Cirja A. Puiu R. Grumezescu A. (2015). Biomedical Applications of Gold Nanoparticles. Curr. Top. Med. Chem. 15 (16), 1605–1613. 10.2174/1568026615666150414144750 25877087

[B15] Chang J. Wang X. Wang J. Li H. Li F. (2019). Nucleic Acid-Functionalized Metal–Organic Framework-Based Homogeneous Electrochemical Biosensor for Simultaneous Detection of Multiple Tumor Biomarkers. ACS Publ. 91 (5), 17. 10.1021/acs.analchem.8b05599 30757896

[B16] Chen B. Mei L. Fan R. Wang Y. Nie C. Tong A. (2021). Facile Construction of Targeted pH-Responsive DNA-Conjugated Gold Nanoparticles for Synergistic Photothermal-Chemotherapy. Chin. Chem. Lett. 32 (5), 1775–1779. 10.1016/j.cclet.2020.12.058

[B17] Chen C. Chen Q. Zhao Q. Liu M. Guo J. (2017). Value of Combined Detection of Serum CEA, CA72-4, CA19-9, CA15-3 and CA12-5 in the Diagnosis of Gastric Cancer. Ann. Clin. Lab. Sci. 47 (3), 260–263. 28667025

[B18] Cho N. H. Byun G. H. Lim Y.-C. Im S. W. Kim H. Lee H.-E. (2020). Uniform Chiral gap Synthesis for High Dissymmetry Factor in Single Plasmonic Gold Nanoparticle. ACS Nano 14 (3), 3595–3602. 10.1021/acsnano.9b10094 32134639

[B19] Christofi T. Baritaki S. Falzone L. Libra M. Zaravinos A. (2019). Current Perspectives in Cancer Immunotherapy. Cancers (Basel) 11 (10), 1472. 10.3390/cancers11101472 PMC6826426 31575023

[B20] Cui S. Cheng Z. Qin W. Jiang L. (2018). Exosomes as a Liquid Biopsy for Lung Cancer. Lung Cancer 116, 46–54. Elsevier. 10.1016/j.lungcan.2017.12.012 29413050

[B21] del Pilar Rodríguez-Torres M. Díaz-Torres L. Romero-Servin S. (2014). Heparin Assisted Photochemical Synthesis of Gold Nanoparticles and Their Performance as SERS Substrates. Int. J. Mol. Sci. 15 (10), 19239–19252. 10.3390/ijms151019239 25342319 PMC4227271

[B22] Deng Z. Y. Chen K. L. Wu C. H. (2019). Improving the SERS Signals of Biomolecules Using a Stacked Biochip Containing Fe2O3/Au Nanoparticles and a DC Magnetic Field. Sci. Rep. 9 (1), 9566–9568. 10.1038/s41598-019-45879-5 31266975 PMC6606591

[B23] Dinish U. S. Fu C. Y. Soh K. S. Ramaswamy B. Kumar A. Olivo M. (2012). Highly Sensitive SERS Detection of Cancer Proteins in Low Sample Volume Using Hollow Core Photonic crystal Fiber. Biosens. Bioelectron. 33 (1), 293–298. 22265083 10.1016/j.bios.2011.12.056

[B24] Doan V. D. Thieu A. T. Nguyen T. D. Nguyen V. C. Cao X. T. Nguyen T. L. (2020). Biosynthesis of Gold Nanoparticles Using Litsea Cubeba Fruit Extract for Catalytic Reduction of 4-Nitrophenol. J. Nanomater. 2020, 10. 10.1155/2020/4548790

[B25] Dobrowolska P. Krajewska A. Gajda-Rączka M. Bartosewicz B. Nyga P. Jankiewicz B. (2015). Application of Turkevich Method for Gold Nanoparticles Synthesis to Fabrication of SiO2@Au and TiO2@Au Core-Shell Nanostructures. Materials 8, 2849–2862. 10.3390/ma8062849

[B26] Du X. Shao Y. Qin H.-F. Tai Y.-H. Gao H.-J. (2018). ALK- Rearrangement in Non-small-cell Lung Cancer (NSCLC). Thorac. Cancer 9 (4), 423–430. 10.1111/1759-7714.12613 29488330 PMC5879058

[B27] EBSCOhost. 2021 EBSCOhost | 148409394 | CA 27-29: A Valuable Marker for Breast Cancer Management in Correlation with CA 15-3.” [Online]. Available: https://web.b.ebscohost.com/abstract?direct=true&profile=ehost&scope=site&authtype=crawler&jrnl=09739122&AN=148409394&h=Onf%2Bd6Is5av6k48xm5roByHKzximpCC1i8DQq49%2BZ%2BoaTfq%2F2oa4Vpdz6XxDMRWDKo9SgLfYKY1PF01zOuRQVg%3D%3D&crl=c&resultNs=AdminWebAuth&resultLocal=ErrCrlNoProfile&crlhashurl=login.aspx%3Fdirect%3Dtrue%26profile%3Dehost%26scope%3Dsite%26authtype%3Dcrawler%26jrnl%3D09739122%26AN%3D148409394 . [Accessed: 30--Sep-2021].

[B28] El Rassy E. Khaled H. Pavlidis N. (2018). Liquid Biopsy: a New Diagnostic, Predictive and Prognostic Window in Cancers of Unknown Primary. Eur. J. Cancer 105, 28–32. 10.1016/j.ejca.2018.09.035 30388661

[B29] Elahi N. Kamali M. Baghersad M. H. Amini B. (2019). A Fluorescence Nano-Biosensors Immobilization on Iron (MNPs) and Gold (AuNPs) Nanoparticles for Detection of Shigella Spp. Mater. Sci. Eng. C 105, 110113. 10.1016/j.msec.2019.110113 31546438

[B30] Eom G. Hwang A. Kim H. Moon J. Kang H. Jung J. (2021). Ultrasensitive Detection of Ovarian Cancer Biomarker Using Au Nanoplate SERS Immunoassay. Biochip J. 15, 348–355. 10.1007/s13206-021-00031-2

[B31] Farshchi F. Hasanzadeh M. Mokhtarzadeh A. (2020). A Novel Electroconductive Interface Based on Fe3 O4 Magnetic Nanoparticle and Cysteamine Functionalized AuNPs: Preparation and Application as Signal Amplification Element to Minoring of Antigen-Antibody Immunocomplex and Biosensing of Prostate Cancer. J. Mol. Recognit. 33 (4), e2825. 10.1002/jmr.2825 31828877

[B32] Fazleeva R. R. Nasretdinova G. R. Osin Y. N. Samigullina A. I. Gubaidullin A. T. Yanilkin V. V. (2021). An Effective Producing Method of Nanocomposites of Ag, Au, and Pd Nanoparticles with Poly(N-Vinylpyrrolidone) and Nanocellulose. Electrocatalysis 12 (3), 225–237. 10.1007/s12678-021-00645-y

[B33] Focsan M. Craciun A. M. Potara M. Leordean C. Vulpoi A. Maniu D. (2017). Flexible and Tunable 3D Gold Nanocups Platform as Plasmonic Biosensor for Specific Dual LSPR-SERS Immuno-Detection. Sci. Rep. 7, 14240. 10.1038/s41598-017-14694-1 29079816 PMC5660151

[B34] Folorunso A. Akintelu S. Oyebamiji A. K. Ajayi S. Abiola B. Abdusalam I. (2019). Biosynthesis, Characterization and Antimicrobial Activity of Gold Nanoparticles from Leaf Extracts of Annona Muricata. J. Nanostruct Chem. 9 (2), 111–117. 10.1007/s40097-019-0301-1

[B35] Gálvez-Vergara A. Fresco-Cala B. Cárdenas S. (2020). Switchable Pickering Emulsions Stabilized by Polystyrene-Modified Magnetic Nanoparticles. Colloids Surf. A: Physicochemical Eng. Aspects 606, 125462. 10.1016/j.colsurfa.2020.125462

[B36] García G. R. d’Orlyé F. Richard C. Mignet N. Varenne A. (2021). Electrokinetic Elucidation of the Interactions between Persistent Luminescent Nanoprobes and the Binary Apolipoprotein-E/albumin Protein System. Analyst 146 (17), 5245–5254. 34296726 10.1039/d1an00781e

[B37] Gric T. Hess O. (2019). Active Optical Metamaterials. Phenom. Opt. Metamaterials 59, 187–261. 10.1016/B978-0-444-63379-8.00001-5

[B38] Grubisha D. S. Lipert R. J. Park H.-Y. Driskell J. Porter M. D. (2003). Femtomolar Detection of Prostate-specific Antigen: an Immunoassay Based on Surface-Enhanced Raman Scattering and Immunogold Labels. Anal. Chem. 75 (21), 5936–5943. 10.1021/ac034356f 14588035

[B39] Grzelczak M. Pérez-Juste J. Mulvaney P. Liz-Marzán L. M. (2008). Shape Control in Gold Nanoparticle Synthesis. Chem. Soc. Rev. 37 (9), 1783–1791. 10.1039/b711490g 18762828

[B40] Gupta A. Mathur R. Singh S. Bag N. Khan U. A. Ahmad F. J. (2021). 99mTc-Methionine Gold Nanoparticles as a Promising Biomaterial for Enhanced Tumor Imaging. J. Pharm. Sci. 110 (2), 888–897. 10.1016/j.xphs.2020.11.008 33212161

[B41] Haber D. A. Velculescu V. E. (2014). Blood-Based Analyses of Cancer: Circulating Tumor Cells and Circulating Tumor DNA. Cancer Discov. 4 (6), 650–661. 10.1158/2159-8290.cd-13-1014 24801577 PMC4433544

[B42] Hawkes N. (2019). Cancer Survival Data Emphasise Importance of Early Diagnosis. BMJ 364, l408. 10.1136/bmj.l408 30683652

[B43] He A. Liu T.-C. Dong Z.-N. Ren Z.-Q. Hou J.-Y. Li M. (2013). A Novel Immunoassay for the Quantization of CYFRA 21-1 in Human Serum. J. Clin. Lab. Anal. 27 (4), 277–283. 10.1002/jcla.21597 23852784 PMC6807541

[B44] Hong S. Li X. (2013). Optimal Size of Gold Nanoparticles for Surface-Enhanced Raman Spectroscopy under Different Conditions. J. Nanomater. 2013. 10.1155/2013/790323

[B45] Hu L. Zhang L. Zhou Y. Meng G. Yu Y. Yao W. (2018). Chitosan-Stabilized Gold Nano Composite Modified Glassy Carbon Electrode for Electrochemical Sensing Trace Hg2+ in Practice. J. Electrochem. Soc. 165 (16), B900–B905. 10.1149/2.1101816jes

[B46] Hu P.-J. Chen M.-Y. Wu M.-S. Lin Y.-C. Shih P.-H. Lai C.-H. (2019). Clinical Evaluation of CA72-4 for Screening Gastric Cancer in a Healthy Population: A Multicenter Retrospective Study. Cancers 11 (5), 733. 10.3390/cancers11050733 PMC6562516 31137895

[B47] Huang X. Jain P. K. El-Sayed I. H. El-Sayed M. A. (2007). Gold Nanoparticles: Interesting Optical Properties and Recent Applications in Cancer Diagnostics and Therapy. Nanomedicine 2 (5), 681–693. 10.2217/17435889.2.5.681 17976030

[B48] Irfana Ishaq Sindhu I. I. Nida Noor N. Raheela Mansoor R. (2021). Choriocarcinoma Syndrome: A Rare Presentation of Testicular Germ Cell Tumour. J. Pak. Med. Assoc. 71 (8), 2090–2092. 10.47391/jpma.234 34418038

[B49] Jazayeri M. H. Aghaie T. Nedaeinia R. Manian M. Nickho H. (2020). Rapid Noninvasive Detection of Bladder Cancer Using Survivin Antibody-Conjugated Gold Nanoparticles (GNPs) Based on Localized Surface Plasmon Resonance (LSPR). Cancer Immunol. Immunother. 69 (9), 1833–1840. 10.1007/s00262-020-02559-y 32350593 PMC11027635

[B50] Jewrajka S. K. Chatterjee U. (2006). Block Copolymer Mediated Synthesis of Amphiphilic Gold Nanoparticles in Water and an Aqueous Tetrahydrofuran Medium: An Approach for the Preparation of Polymer-Gold Nanocomposites. J. Polym. Sci. A. Polym. Chem. 44 (6), 1841–1854. 10.1002/pola.21293

[B51] Jiang G. Wang L. Chen W. (2007). Studies on the Preparation and Characterization of Gold Nanoparticles Protected by Dendrons. Mater. Lett. 61 (1), 278–283. 10.1016/j.matlet.2006.04.110

[B52] Jiang L.-P. Xu S. Zhu J.-M. Zhang J.-R. Zhu J.-J. Chen H.-Y. (2004). Ultrasonic-assisted Synthesis of Monodisperse Single-Crystalline Silver Nanoplates and Gold Nanorings. Inorg. Chem. 43 (19), 5877–5883. 10.1021/ic049529d 15360236

[B53] Khalil I. Yehye W. A. Julkapli N. M. Rahmati S. Sina A. A. I. Basirun W. J. (2019). Graphene Oxide and Gold Nanoparticle Based Dual Platform with Short DNA Probe for the PCR Free DNA Biosensing Using Surface-Enhanced Raman Scattering. Biosens. Bioelectron. 131, 214–223. 10.1016/j.bios.2019.02.028 30844598

[B54] Khatua A. Priyadarshini E. Rajamani P. Patel A. Kumar J. Naik A. (2020). Phytosynthesis, Characterization and Fungicidal Potential of Emerging Gold Nanoparticles Using Pongamia Pinnata Leave Extract: A Novel Approach in Nanoparticle Synthesis. J. Clust. Sci. 31 (1), 125–131. 10.1007/s10876-019-01624-6

[B55] Kim H.-M. Hong Jeong D. Lee H.-Y. Park J.-H. Lee S.-K. (2019). Improved Stability of Gold Nanoparticles on the Optical Fiber and Their Application to Refractive index Sensor Based on Localized Surface Plasmon Resonance. Opt. Laser Techn. 114, 171–178. 10.1016/j.optlastec.2019.02.002

[B56] Kim H.-M. Jeong D. H. Lee H.-Y. Park J.-H. Lee S.-K. (2021). Design and Validation of Fiber Optic Localized Surface Plasmon Resonance Sensor for Thyroglobulin Immunoassay with High Sensitivity and Rapid Detection. Sci. Rep. 11 (1), 1–9. 10.1038/s41598-021-95375-y 34362953 PMC8346482

[B57] Kim H. Lee D. (2018). Near-Infrared-Responsive Cancer Photothermal and Photodynamic Therapy Using Gold Nanoparticles. Polymers 10 (9), 961. 10.3390/polym10090961 PMC6403910 30960886

[B58] Koushki E. Mirzaei Mohammadabadi F. Baedi J. Ghasedi A. (2020). The Effects of Glucose and Glucose Oxidase on the Uv-Vis Spectrum of Gold Nanoparticles: A Study on Optical Biosensor for Saliva Glucose Monitoring. Photodiagnosis Photodynamic Ther. 30, 101771. 10.1016/j.pdpdt.2020.101771 32311543

[B59] Kuntamung K. Sangthong P. Jakmunee J. Ounnunkad K. (2021). A Label-free Immunosensor for the Detection of a New Lung Cancer Biomarker, GM2 Activator Protein, Using a Phosphomolybdic Acid/polyethyleneimine Coated Gold Nanoparticle Composite. Analyst 146 (7), 2203–2211. 10.1039/d0an02149k 33595007

[B60] Kwaan H. C. Lindholm P. F. (2019). Fibrin and Fibrinolysis in Cancer. Semin. Thromb. Hemost. 45 (4), 413–422. 10.1055/s-0039-1688495 31041799

[B61] Lane R. E. Korbie D. Hill M. M. Trau M. (2018). Extracellular Vesicles as Circulating Cancer Biomarkers: Opportunities and Challenges. Clin. Transl. Med. 7 (1), 14. 10.1186/s40169-018-0192-7 29855735 PMC5981152

[B62] Lee Y. J. Ahn E. Y. Park Y. (2019). Shape-dependent Cytotoxicity and Cellular Uptake of Gold Nanoparticles Synthesized Using green tea Extract. Nanoscale Res. Lett. 14 (1), 129. 10.1186/s11671-019-2967-1 30976946 PMC6459462

[B63] Lewis J. M. Vyas A. D. Qiu Y. Messer K. S. White R. Heller M. J. (2018). Integrated Analysis of Exosomal Protein Biomarkers on Alternating Current Electrokinetic Chips Enables Rapid Detection of Pancreatic Cancer in Patient Blood. ACS Nano 12 (4), 3311–3320. 10.1021/acsnano.7b08199 29570265

[B64] Li H. Hu W. Hassan M. M. Zhang Z. Chen Q. (2019). A Facile and Sensitive SERS-Based Biosensor for Colormetric Detection of Acetamiprid in green tea Based on Unmodified Gold Nanoparticles. Food Measure 13 (1), 259–268. 10.1007/s11694-018-9940-z

[B65] Li K. Liu G. Wu Y. Hao P. Zhou W. Zhang Z. (2014). Gold Nanoparticle Amplified Optical Microfiber Evanescent Wave Absorption Biosensor for Cancer Biomarker Detection in Serum. Talanta 120, 419–424. 10.1016/j.talanta.2013.11.085 24468391

[B66] Li M. Cushing S. K. Zhang J. Suri S. Evans R. Petros W. P. (2013). Three-Dimensional Hierarchical Plasmonic Nano-Architecture Enhanced Surface-Enhanced Raman Scattering Immunosensor for Cancer Biomarker Detection in Blood Plasma. ACS Nano 7 (6), 4967–4976. 10.1021/nn4018284 23659430 PMC3732798

[B67] Li M. Kang J. W. Sukumar S. Dasari R. R. Barman I. (2015). Multiplexed Detection of Serological Cancer Markers with Plasmon-Enhanced Raman Spectro-Immunoassay. Chem. Sci. 6 (7), 3906–3914. 10.1039/c5sc01054c 26405519 PMC4577055

[B68] Li W. Li C. Zhou T. Liu X. Liu X. Li X. (2017). Role of Exosomal Proteins in Cancer Diagnosis. Mol. Cancer 16 (1), 145. 10.1186/s12943-017-0706-8 28851367 PMC5576100

[B69] Li X. Dai D. Chen B. Tang H. Xie X. Wei W. (2018). Clinicopathological and Prognostic Significance of Cancer Antigen 15-3 and Carcinoembryonic Antigen in Breast Cancer: a Meta-Analysis Including 12,993 Patients. Dis. Markers 2018, 1–15. 10.1155/2018/9863092 PMC5954898 29854028

[B70] Lin L.-K. Uzunoglu A. Stanciu L. A. Lin L. Uzunoglu A. Stanciu L. A. (2018). Aminolated and Thiolated PEG-Covered Gold Nanoparticles with High Stability and Antiaggregation for Lateral Flow Detection of Bisphenol A. Small 14 (10), 1702828. 10.1002/smll.201702828 29280330

[B71] Liu D. Huang X. Wang Z. Jin A. Sun X. Zhu L. (2013). Gold Nanoparticle-Based Activatable Probe for Sensing Ultralow Levels of Prostate-specific Antigen. ACS Nano 7 (6), 5568–5576. 10.1021/nn401837q 23683064 PMC3696512

[B72] Liu Y. Fu W. Xu Z. Zhang L. Sun T. Du M. (2021). pH-Driven Reversible Assembly and Disassembly of Colloidal Gold Nanoparticles. Front. Chem. 9, 183. 10.3389/fchem.2021.675491 PMC8116534 33996769

[B73] Lomelí-Marroquín D. Medina Cruz D. Nieto-Argüello A. Vernet Crua A. Chen J. Torres-Castro A. (2019). Starch-mediated Synthesis of Mono- and Bimetallic Silver/gold Nanoparticles as Antimicrobial and Anticancer Agents. Int. J. Nanomedicine 14, 2171. 10.2147/IJN.S192757 30988615 PMC6443225

[B74] Lu J. Song Y. Lei F. Du X. Huo Y. Xu S. (2020). Electric Field-Modulated Surface Enhanced Raman Spectroscopy by PVDF/Ag Hybrid. Sci. Rep. 10 (1), 5269. 10.1038/s41598-020-62251-0 32210311 PMC7093541

[B75] Ma Y. Liu H. Chen Y. Gu C. Wei G. Jiang T. (2020). Improved Lateral Flow Strip Based on Hydrophilic−hydrophobic SERS Substrate for Ultra−sensitive and Quantitative Immunoassay. Appl. Surf. Sci. 529, 147121. 10.1016/j.apsusc.2020.147121

[B76] Mafuné F. Kohno J. Takeda Y. (2001). Formation of Gold Nanoparticles by Laser Ablation in Aqueous Solution of Surfactant. J. Phys. Chem. B. 105 (22), 5114–5120. 10.1021/jp0037091

[B77] Mahani M. Alimohamadi F. Torkzadeh-Mahani M. Hassani Z. Khakbaz F. Divsar F. (2021). LSPR Biosensing for the Early-Stage Prostate Cancer Detection Using Hydrogen Bonds between PSA and Antibody: Molecular Dynamic and Experimental Study. J. Mol. Liquids 324, 114736. 10.1016/j.molliq.2020.114736

[B78] Mahato K. Nagpal S. Shah M. A. Srivastava A. Maurya P. K. Roy S. (2019). Gold Nanoparticle Surface Engineering Strategies and Their Applications in Biomedicine and Diagnostics. 3 Biotech. 9 (2), 57. 10.1007/s13205-019-1577-z PMC6352626 30729081

[B79] Maity A. De S. K. Chakraborty A. (2021). Interaction of Aromatic Amino Acid-Functionalized Gold Nanoparticles with Lipid Bilayers: Insight into the Emergence of Novel Lipid Corona Formation. J. Phys. Chem. B 125 (8), 2113–2123. 10.1021/acs.jpcb.0c10079 33605726

[B80] Man R. Li C. MacLean M. W. A. Zenkina O. V. Zamora M. T. Saunders L. N. (2018). Ultrastable Gold Nanoparticles Modified by Bidentate N-Heterocyclic Carbene Ligands. J. Am. Chem. Soc. 140 (5), 1576–1579. 10.1021/jacs.7b08516, 29211456

[B81] Matsuyama A. Higashi S. Tanizaki S. Morotomi T. Washio A. Ohsumi T. (2018). Celecoxib Inhibits Osteoblast Differentiation Independent of Cyclooxygenase Activity. Clin. Exp. Pharmacol. Physiol. 45 (1), 75–83. 10.1111/1440-1681.12846 28815657

[B82] Matteini P. Ratto F. Rossi F. Centi S. Dei L. Pini R. (2010). Chitosan Films Doped with Gold Nanorods as Laser-Activatable Hybrid Bioadhesives. Adv. Mater. 22 (38), 4313–4316. 10.1002/adma.201002228 20734385

[B83] Medetalibeyoglu H. Kotan G. Atar N. Yola M. L. (2020). A Novel sandwich-type SERS Immunosensor for Selective and Sensitive Carcinoembryonic Antigen (CEA) Detection. Analytica Chim. Acta 1139, 100–110. 10.1016/j.aca.2020.09.034 33190692

[B84] Medici S. Peana M. Coradduzza D. Zoroddu M. A. (2021). Gold Nanoparticles and Cancer: Detection, Diagnosis and Therapy. Semin. Cancer Biol. 76, 27–37. 10.1016/j.semcancer.2021.06.017 34153434

[B85] Misek D. E. Kim E. H. (2011). Protein Biomarkers for the Early Detection of Breast Cancer. Int. J. Proteomics 2011, 1–9. 10.1155/2011/343582 PMC3195294 22084684

[B86] Moore T. Moody A. Payne T. Sarabia G. Daniel A. Sharma B. (2018). *In Vitro* and *In Vivo* SERS Biosensing for Disease Diagnosis. Biosensors 8 (2), 46. 10.3390/bios8020046 PMC6022968 29751641

[B87] Moskovits M. (2005). Surface-enhanced Raman Spectroscopy: a Brief Retrospective. J. Raman Spectrosc. 36 (6–7), 485–496. 10.1002/jrs.1362

[B88] Muhammad M. Shao C.-s. Huang Q. (2021). Aptamer-functionalized Au Nanoparticles Array as the Effective SERS Biosensor for Label-free Detection of Interleukin-6 in Serum. Sensors Actuators B: Chem. 334, 129607. 10.1016/j.snb.2021.129607

[B89] Nam J.-M. Thaxton C. S. Mirkin C. A. (2003). Nanoparticle-based Bio-Bar Codes for the Ultrasensitive Detection of Proteins. Science 301 (5641), 1884–1886. 10.1126/science.1088755 14512622

[B90] Niederle M. B. Scheuba C. Riss P. Selberherr A. Koperek O. Niederle B. (2020). Early Diagnosis of Medullary Thyroid Cancer: Are Calcitonin Stimulation Tests Still Indicated in the Era of Highly Sensitive Calcitonin Immunoassays? Thyroid 30 (7), 974–984. 10.1089/thy.2019.0785 32056502

[B91] Nolen B. M. Lokshin A. E. (2012). Protein Biomarkers of Ovarian Cancer: the forest and the Trees. Future Oncol. 8 (1), 55–71. 10.2217/fon.11.135 22149035 PMC3312922

[B92] Okitsu K. Yue A. Tanabe S. Matsumoto H. Yobiko Y. (2001). Formation of Colloidal Gold Nanoparticles in an Ultrasonic Field: Control of Rate of Gold(III) Reduction and Size of Formed Gold Particles. Langmuir 17 (25), 7717–7720. 10.1021/la010414l

[B93] Othman H. O. Salehnia F. Fakhri N. Hassan R. Hosseini M. Faizullah A. (2020). A Highly Sensitive Fluorescent Immunosensor for Sensitive Detection of Nuclear Matrix Protein 22 as Biomarker for Early Stage Diagnosis of Bladder Cancer. RSC Adv. 10 (48), 28865–28871. 10.1039/d0ra06191c 35520044 PMC9055858

[B94] Ouellette M. Masse F. Lefebvre-Demers M. Maestracci Q. Grenier P. Millar R. (2018). Insights into Gold Nanoparticles as a Mucoadhesive System. Sci. Rep. 8 (1), 14357. 10.1038/s41598-018-32699-2 30254340 PMC6156509

[B95] Ouhibi A. Raouafi A. Lorrain N. Guendouz M. Raouafi N. Moadhen A. (2021). Functionalized SERS Substrate Based on Silicon Nanowires for Rapid Detection of Prostate Specific Antigen. Sensors Actuators B: Chem. 330, 129352. 10.1016/j.snb.2020.129352

[B96] Palermo G. Rippa M. Conti Y. Vestri A. Castagna R. Fusco G. (2021). Plasmonic Metasurfaces Based on Pyramidal Nanoholes for High-Efficiency SERS Biosensing. ACS Appl. Mater. Inter. 13 (36), 43715–43725. 10.1021/acsami.1c12525 PMC8447193 34469103

[B97] Peng N.-J. Hu C. Chiu Y.-L. Yu C.-C. Li C.-J. Sheu J. J.-C. (2020). Detection of Recurrent Cervical Cancer and Prediction of its Patient Survival with Serum Squamous-Cell Carcinoma-Antigen and 2-[18F] Fluoro-2-Deoxy-D-Glucose-Positron Emission Tomography/Computed Tomography. Diagnostics 10 (9), 657. 10.3390/diagnostics10090657 PMC7555056 32878219

[B98] Polte J. Ahner T. T. Delissen F. Sokolov S. Emmerling F. Thünemann A. F. (2010). Mechanism of Gold Nanoparticle Formation in the Classical Citrate Synthesis Method Derived from Coupled *In Situ* XANES and SAXS Evaluation. J. Am. Chem. Soc. 132 (4), 1296–1301. 10.1021/ja906506j 20102229

[B99] Qamar M. Abbas G. Afzaal M. Naz M. Y. Ghuffar A. Irfan M. (2022). Gold Nanorods for Doxorubicin Delivery: Numerical Analysis of Electric Field Enhancement, Optical Properties and Drug Loading/Releasing Efficiency. Mater 15 (5), 1764. 10.3390/ma15051764 PMC8911263 35268995

[B100] Ramírez S. H. A. García Casillas P. González C. C. (2020). Seed-mediated Synthesis and PEG Coating of Gold Nanoparticles for Controlling Morphology and Sizes. MRS Adv. 5 (63), 3353–3360. 10.1557/adv.2020.416

[B101] Retout M. Valkenier H. Triffaux E. Doneux T. Bartik K. Bruylants G. (2016). Rapid and Selective Detection of Proteins by Dual Trapping Using Gold Nanoparticles Functionalized with Peptide Aptamers. ACS Sens. 1 (7), 929–933. 10.1021/acssensors.6b00229

[B102] Rojalin T. Koster H. J. Liu J. Mizenko R. R. Tran D. Wachsmann-Hogiu S. (2020). Hybrid Nanoplasmonic Porous Biomaterial Scaffold for Liquid Biopsy Diagnostics Using Extracellular Vesicles. ACS Sens. 5 (9), 2820–2833. 10.1021/acssensors.0c00953 32935542 PMC7522966

[B103] Romeo F. Seropian I. (2021). Additive Prognostic Value of Carbohydrate Antigen‐125 over Frailty in Patients Undergoing Transcatheter Aortic Valve Replacement. Wiley Online Libr. 97 (2), E263–E273. 10.1002/ccd.29067 32597028

[B104] Ruff J. Hassan N. Morales-Zavala F. Steitz J. Araya E. Kogan M. J. (2018). CLPFFD-PEG Functionalized NIR-Absorbing Hollow Gold Nanospheres and Gold Nanorods Inhibit β-amyloid Aggregation. J. Mater. Chem. B 6 (16), 2432–2443. 10.1039/c8tb00655e 32254460

[B105] Saeed A. A. Sánchez J. L. A. O'Sullivan C. K. Abbas M. N. (2017). DNA Biosensors Based on Gold Nanoparticles-Modified Graphene Oxide for the Detection of Breast Cancer Biomarkers for Early Diagnosis. Bioelectrochemistry 118, 91–99. 10.1016/j.bioelechem.2017.07.002 28802177

[B106] Salabat A. Mirhoseini F. (2018). A Novel and Simple Microemulsion Method for Synthesis of Biocompatible Functionalized Gold Nanoparticles. J. Mol. Liquids 268, 849–853. 10.1016/j.molliq.2018.07.112

[B107] Sanders M. Lin Y. Wei J. Bono T. Lindquist R. G. (2014). An Enhanced LSPR Fiber-Optic Nanoprobe for Ultrasensitive Detection of Protein Biomarkers. Biosens. Bioelectron. 61, 95–101. 10.1016/j.bios.2014.05.009 24858997

[B108] Sanzhakov M. A. Kudinov V. A. Baskaev K. K. Morozevich G. E. Stepanova D. S. Torkhovskaya T. I. (2021). Composite Phospholipid-Gold Nanoparticles with Targeted Fragment for Tumor Imaging. Biomed. Pharmacother. 142, 111985. 10.1016/j.biopha.2021.111985 34352716

[B109] Sau T. K. Murphy C. J. (2004). Room Temperature, High-Yield Synthesis of Multiple Shapes of Gold Nanoparticles in Aqueous Solution. J. Am. Chem. Soc. 126 (28), 8648–8649. 10.1021/ja047846d 15250706

[B110] Sau T. K. Pal A. Jana N. R. Wang Z. L. Pal T. (2001). Size Controlled Synthesis of Gold Nanoparticles Using Photochemically Prepared Seed Particles. J. Nanoparticle Res. 3 (4), 257–261. 10.1023/a:1017567225071

[B111] Schulz F. Homolka T. Bastús N. G. Puntes V. Weller H. Vossmeyer T. (2014). Little Adjustments Significantly Improve the Turkevich Synthesis of Gold Nanoparticles. Langmuir 30 (35), 10779–10784. 10.1021/la503209b 25127436

[B112] Shah P. A. Onken A. Ishtiaq R. Maqsood M. H. Patel S. S. Campoverde Reyes K. J. (2020). A Challenging Case of Alpha-Fetoprotein-Result Discrepancies in a Patient with Chronic Hepatitis B. Gastroenterol. Rep. 8 (6), 484–486. 10.1093/gastro/goaa052 PMC7793204 33447392

[B113] Shedbalkar U. Singh R. Wadhwani S. Gaidhani S. Chopade B. A. (2014). Microbial Synthesis of Gold Nanoparticles: Current Status and Future Prospects. Adv. Colloid Interf. Sci. 209, 40–48. 10.1016/j.cis.2013.12.011 24456802

[B114] Shenhar R. Norsten T. B. Rotello V. M. (2005). Polymer-Mediated Nanoparticle Assembly: Structural Control and Applications. Adv. Mater. 17 (6), 657–669. 10.1002/adma.200401291

[B115] Shin H. Jeong H. Park J. Hong S. Choi Y. (2018). Correlation between Cancerous Exosomes and Protein Markers Based on Surface-Enhanced Raman Spectroscopy (SERS) and Principal Component Analysis (PCA). ACS Sens. 3 (12), 2637–2643. 10.1021/acssensors.8b01047 30381940

[B116] Singh R. Azad A. K. Zhang W. (2013). “Resonant Field Enhancement of Terahertz Waves in Subwavelength Plasmonic Structures,” in Handb. Terahertz Technol. Imaging, Sens. Commun. (Sawston, United Kingdom: Woodhead Publishing), 272–294. 10.1533/9780857096494.2.272

[B117] Song Y. Sun J. Zhao S. Gao F. Yuan H. Sun B. (2022). Based Lateral Flow Immunosensor for Ultrasensitive and Selective Surface-Enhanced Raman Spectroscopy Stroke Biomarkers Detection. Appl. Surf. Sci. 571, 151153. 10.1016/j.apsusc.2021.151153

[B118] Subki A. Alghamdi T. Butt N. Alqazlan M. S. Alkahtani A. M. Aziz M. A. (2021). CEA and CA19-9 Levels and KRAS Mutation Status as Biomarkers for Colorectal Cancer. Clin. Oncol. 6, 1802.

[B119] Surinova S. Schiess R. Hüttenhain R. Cerciello F. Wollscheid B. Aebersold R. (2011). On the Development of Plasma Protein Biomarkers. J. Proteome Res. 10 (1), 5–16. 10.1021/pr1008515 21142170

[B120] Thakkar K. N. Mhatre S. S. Parikh R. Y. (2010). Biological Synthesis of Metallic Nanoparticles. Nanomedicine: Nanotechnology, Biol. Med. 6 (2), 257–262. 10.1016/j.nano.2009.07.002 19616126

[B121] Thiruppathi R. Mishra S. Ganapathy M. Padmanabhan P. Gulyás B. (2017). Nanoparticle Functionalization and its Potentials for Molecular Imaging. Adv. Sci. 4 (3), 1600279. 10.1002/advs.201600279 PMC5357986 28331783

[B122] Tianimoghadam S. Salabat A. (2018). A Microemulsion Method for Preparation of Thiol-Functionalized Gold Nanoparticles. Particuology 37, 33–36. 10.1016/j.partic.2017.05.007

[B123] Tkac J. Gajdosova V. Hroncekova S. Bertok T. Hires M. Jane E. (2019). Prostate-specific Antigen Glycoprofiling as Diagnostic and Prognostic Biomarker of Prostate Cancer. Interf. Focus. 9 (2), 20180077. 10.1098/rsfs.2018.0077 PMC6388024 30842876

[B124] Tommalieh M. J. Ibrahium H. A. Awwad N. S. Menazea A. A. (2020). Gold Nanoparticles Doped Polyvinyl Alcohol/Chitosan Blend via Laser Ablation for Electrical Conductivity Enhancement. J. Mol. Struct. 1221, 128814. 10.1016/j.molstruc.2020.128814

[B125] TunÇ İ. Susapto H. H. (2019). Label-Free Detection of Ovarian Cancer Antigen CA125 by Surface Enhanced Raman Scattering. J. Nanosci. Nanotechnol. 20 (3), 1358–1365. 10.1166/jnn.2020.17141 31492295

[B126] Vijaya Kumar P. Mary Jelastin Kala S. Prakash K. S. (2019). Green Synthesis of Gold Nanoparticles Using Croton Caudatus Geisel Leaf Extract and Their Biological Studies. Mater. Lett. 236, 19–22. 10.1016/j.matlet.2018.10.025

[B127] Vines J. B. Yoon J. H. Ryu N. E. Lim D. J. Park H. (2019). Gold Nanoparticles for Photothermal Cancer Therapy. Front. Chem. 7, 167. 10.3389/fchem.2019.00167 31024882 PMC6460051

[B128] Vorontsov A. V. Tsybulya S. V. (2018). Influence of Nanoparticles Size on XRD Patterns for Small Monodisperse Nanoparticles of Cu0 and TiO2 Anatase. Ind. Eng. Chem. Res. 57 (7), 2526–2536. 10.1021/acs.iecr.7b04480

[B129] Wahab A. Karim A. Asmawati A. Sutapa I. (2018). Bio-synthesis of Gold Nanoparticles through Bioreduction Using the Aqueous Extract of Muntingia calabura L. Leaf. Orient J. Chem. 34 (1), 401–409. 10.13005/ojc/340143

[B130] Wang X. Li G. Chen T. Yang M. Zhang Z. Wu T. (2008). Polymer-encapsulated Gold-Nanoparticle Dimers: Facile Preparation and Catalytical Application in Guided Growth of Dimeric ZnO-Nanowires. Nano Lett. 8 (9), 2643–2647. 10.1021/nl080820q 18672944

[B131] Wang Y. Quinsaat J. E. Q. Ono T. Maeki M. Tokeshi M. Isono T. (2020). Enhanced Dispersion Stability of Gold Nanoparticles by the Physisorption of Cyclic Poly(ethylene Glycol). Nat. Commun. 11 (1), 6089. 10.1038/s41467-020-19947-8 33257670 PMC7705015

[B132] Wang Y. Seidel M. (2021). Strategy for Fast Manufacturing of 3D Hydrodynamic Focusing Multilayer Microfluidic Chips and its Application for Flow-Based Synthesis of Gold Nanoparticles. Microfluid. Nanofluidics 25 (8), 1–10. 10.1007/s10404-021-02463-6

[B133] Yang H. Tian T. Wu D. Guo D. Lu J. (2018). Prevention and Treatment Effects of Edible Berries for Three Deadly Diseases: Cardiovascular Disease, Cancer and Diabetes. Crit. Rev. Food Sci. Nutr. 59 (12), 1903–1912. 10.1080/10408398.2018.1432562 29381386

[B134] You B. Robelin P. Tod M. Louvet C. Lotz J.-P. Abadie-Lacourtoisie S. (2020). CA-125 ELIMination Rate Constant K (KELIM) Is a Marker of Chemosensitivity in Patients with Ovarian Cancer: Results from the Phase II CHIVA Trial. Clin. Cancer Res. 26 (17), 4625–4632. 10.1158/1078-0432.ccr-20-0054 32209570

[B135] Yu Z. Grasso M. F. Cui X. Silva R. N. Zhang P. (2020). Sensitive and Label-free SERS Detection of Single-Stranded DNA Assisted by Silver Nanoparticles and Gold-Coated Magnetic Nanoparticles. ACS Appl. Bio Mater. 3 (5), 2626–2632. 10.1021/acsabm.9b01218 35025396

[B136] Yuan H. Ji W. Chu S. Liu Q. Qian S. Guang J. (2019). Mercaptopyridine-Functionalized Gold Nanoparticles for Fiber-Optic Surface Plasmon Resonance Hg2+ Sensing. ACS Sens. 4 (3), 704–710. 10.1021/acssensors.8b01558 30785267

[B137] Zang R. Li Y. Jin R. Wang X. Lei Y. Che Y. (2019). Enhancement of Diagnostic Performance in Lung Cancers by Combining CEA and CA125 with Autoantibodies Detection. Taylor Fr 8 (10), e1625689. 10.1080/2162402X.2019.1625689 PMC6791432 31646071

[B138] Zhang H. Wang Z. Wang F. Zhang Y. Wang H. Liu Y. (2020). *In Situ* Formation of Gold Nanoparticles Decorated Ti3C2 MXenes Nanoprobe for Highly Sensitive Electrogenerated Chemiluminescence Detection of Exosomes and Their Surface Proteins. Anal. Chem. 92 (7), 5546–5553. 10.1021/acs.analchem.0c00469 32186362

[B139] Zhang L. Fan C. Liu M. Liu F. Bian S. Du S. (2018). Biominerized Gold-Hemin@MOF Composites with Peroxidase-like and Gold Catalysis Activities: A High-Throughput Colorimetric Immunoassay for Alpha-Fetoprotein in Blood by ELISA and Gold-Catalytic Silver Staining. Sensors Actuators B: Chem. 266, 543–552. 10.1016/j.snb.2018.03.153

[B140] Zhang P. Xiao J. Ruan Y. Zhang Z. Zhang X. (2020). Monitoring Value of Serum HER2 as a Predictive Biomarker in Patients with Metastatic Breast Cancer. Cancer Manag. Res. 12, 4667–4675. 10.2147/cmar.s254897 32606958 PMC7308125

[B141] Zhang Y. Li L. Gao Y. Wang X. Sun L. Ji W. (2020). Nitrosonaphthol Reaction-Assisted SERS Assay for Selective Determination of 5-Hydroxyindole-3-Acetic Acid in Human Urine. Analytica Chim. Acta 1134, 34–40. 10.1016/j.aca.2020.08.020 33059864

[B142] Zhao Q. Zhan T. Deng Z. Li Q. Liu Y. Yang S. (2018). Glycan Analysis of Colorectal Cancer Samples Reveals Stage-dependent Changes in CEA Glycosylation Patterns. Clin. Proteomics 15 (1), 9. 10.1186/s12014-018-9182-4 29507546 PMC5834848

[B143] Zheng X. Chen S. Li L. Liu X. Liu X. Dai S. (2018). Evaluation of HE4 and TTR for Diagnosis of Ovarian Cancer: Comparison with CA-125. J. Gynecol. Obstet. Hum. Reprod. 47 (6), 227–230. 10.1016/j.jogoh.2018.03.010 29609043

[B144] Zhou C. Yang Y. Li H. Gao F. Song C. Yang D. (2020). Programming Surface-Enhanced Raman Scattering of DNA Origami-Templated Metamolecules. Nano Lett. 20 (5), 3155–3159. 10.1021/acs.nanolett.9b05161 32286079

[B145] Zhou Y. Wang C. Y. Zhu Y. R. Chen Z. Y. (1999). A Novel Ultraviolet Irradiation Technique for Shape-Controlled Synthesis of Gold Nanoparticles at Room Temperature. Chem. Mater. 11 (9), 2310–2312. 10.1021/cm990315h

[B146] Zhu L. Gharib M. Becker C. Zeng Y. Ziefuß A. R. Chen L. (2020). Synthesis of Fluorescent Silver Nanoclusters: Introducing Bottom-Up and Top-Down Approaches to Nanochemistry in a Single Laboratory Class. J. Chem. Educ. 97 (1), 239–243. 10.1021/acs.jchemed.9b00342

